# Long COVID – a critical disruption of cholinergic neurotransmission?

**DOI:** 10.1186/s42234-025-00167-8

**Published:** 2025-02-27

**Authors:** Marco Leitzke, Donald Troy Roach, Swen Hesse, Peter Schönknecht, Georg-Alexander Becker, Michael Rullmann, Bernhardt Sattler, Osama Sabri

**Affiliations:** 1 Department of Nuclear Medicine, University of Leipzig Medical Centre, Leipzig, 04103 Germany; 2 Department of Anesthesiology, Intensive Care Medicine, Pain- and Palliative Therapy Helios Clinics, Colditzer Straße 48, Leisnig, 04703 Germany; 3 School of Comillas University, Renegade Research, Madrid, 28015 Spain; 4 Department of Psychiatry and Neurology Altscherbitz, Schkeuditz, 04435 Germany; 5 Outpatient Department for Forensic-Psychiatric Research, University of Leipzig, Leipzig, 04103 Germany

**Keywords:** Long COVID, Cholinergic neurotransmission, Nicotinic acetylcholine receptors, Low dose transdermal nicotine, Flubatine, Spike glycoprotein

## Abstract

**Background:**

Following the COVID-19 pandemic, there are many chronically ill Long COVID (LC) patients with different symptoms of varying degrees of severity. The pathological pathways of LC remain unclear until recently and make identification of path mechanisms and exploration of therapeutic options an urgent challenge. There is an apparent relationship between LC symptoms and impaired cholinergic neurotransmission.

**Methods:**

This paper reviews the current literature on the effects of blocked nicotinic acetylcholine receptors (nAChRs) on the main affected organ and cell systems and contrasts this with the unblocking effects of the alkaloid nicotine. In addition, mechanisms are presented that could explain the previously unexplained phenomenon of post-vaccination syndrome (PVS). The fact that not only SARS-CoV-2 but numerous other viruses can bind to nAChRs is discussed under the assumption that numerous other post-viral diseases and autoimmune diseases (ADs) may also be due to impaired cholinergic transmission. We also present a case report that demonstrates changes in cholinergic transmission, specifically, the availability of α4β2 nAChRs by using (-)-[^18^F]Flubatine whole-body positron emission tomography (PET) imaging of cholinergic dysfunction in a LC patient along with a significant neurological improvement before and after low-dose transcutaneous nicotine (LDTN) administration. Lastly, a descriptive analysis and evaluation were conducted on the results of a survey involving 231 users of LDTN.

**Results:**

A substantial body of research has emerged that offers a compelling explanation for the phenomenon of LC, suggesting that it can be plausibly explained because of impaired nAChR function in the human body. Following a ten-day course of transcutaneous nicotine administration, no enduring neuropathological manifestations were observed in the patient. This observation was accompanied by a significant increase in the number of free ligand binding sites (LBS) of nAChRs, as determined by (-)-[^18^F]Flubatine PET imaging. The analysis of the survey shows that the majority of patients (73.5%) report a significant improvement in the symptoms of their LC/MEF/CFS disease as a result of LDTN.

**Conclusions:**

In conclusion, based on current knowledge, LDTN appears to be a promising and safe procedure to relieve LC symptoms with no expected long-term harm.

**Supplementary Information:**

The online version contains supplementary material available at 10.1186/s42234-025-00167-8.

## Introduction

The WHO declared the SARS-CoV-2 pandemic over on May 5, 2023 (Lenharo 2023). In the meantime, global healthcare systems are facing a complex of symptoms as a post-viral syndrome with a cumulative global incidence of around 400 million people (Al-Aly 2024). The appearance of the syndrome, referred to as long COVID (LC) (O'Mahoney 2023) or post-acute sequalae of COVID-19 (PASC) (Swank 2024) is reported to be between 10 and 30% of outpatient SARS-CoV-2 infections and 50–70% of hospitalized COVID-19 cases (Ceban 2022). The causes of this extremely diverse clinical picture (Table 1) are still unclear. Until recently, most of the commonly observed pathological findings could only be managed symptomatically. An etiopathogenetic pathway has not been identified, and thus a curative therapeutic approach has not yet been established. Interestingly, there is a high degree of symptomatic concordance with the well-characterized symptom complex of Myalgic Encephalomyelitis /chronic fatigue syndrome (ME/CFS) (Komaroff 2023), which is also thought to have a post-viral/post-infection genesis (Pricoco 2024). In addition, there are increasing reports that, in rare cases, a very similar clinical picture also develops after vaccination against SARS-CoV-2 infection (Elsaid 2023; Altmann et al. 2023), which is referred to as post-vaccination syndrome (PVS) (Krumholz 2023). Finally, reactivation of various persistent viral diseases (Altmann et al. 2023) (EBV (Altmann et al. 2023), CMV (Altmann et al. 2023), HIV (Peluso 2023) as well as IAV (Kim 2023), IBV (Kim 2023), VZV (Kim 2023), HHV-1 (Kim 2023), HHV-2 (Kim 2023), HHV-6 (Kim 2023), HHV-7 (Kim 2023), HHV-8 (Kim 2023), HBV (Kim 2023), and Parvovirus B19 (Kim 2023) has been described in association with long COVID.

**Table 1:**
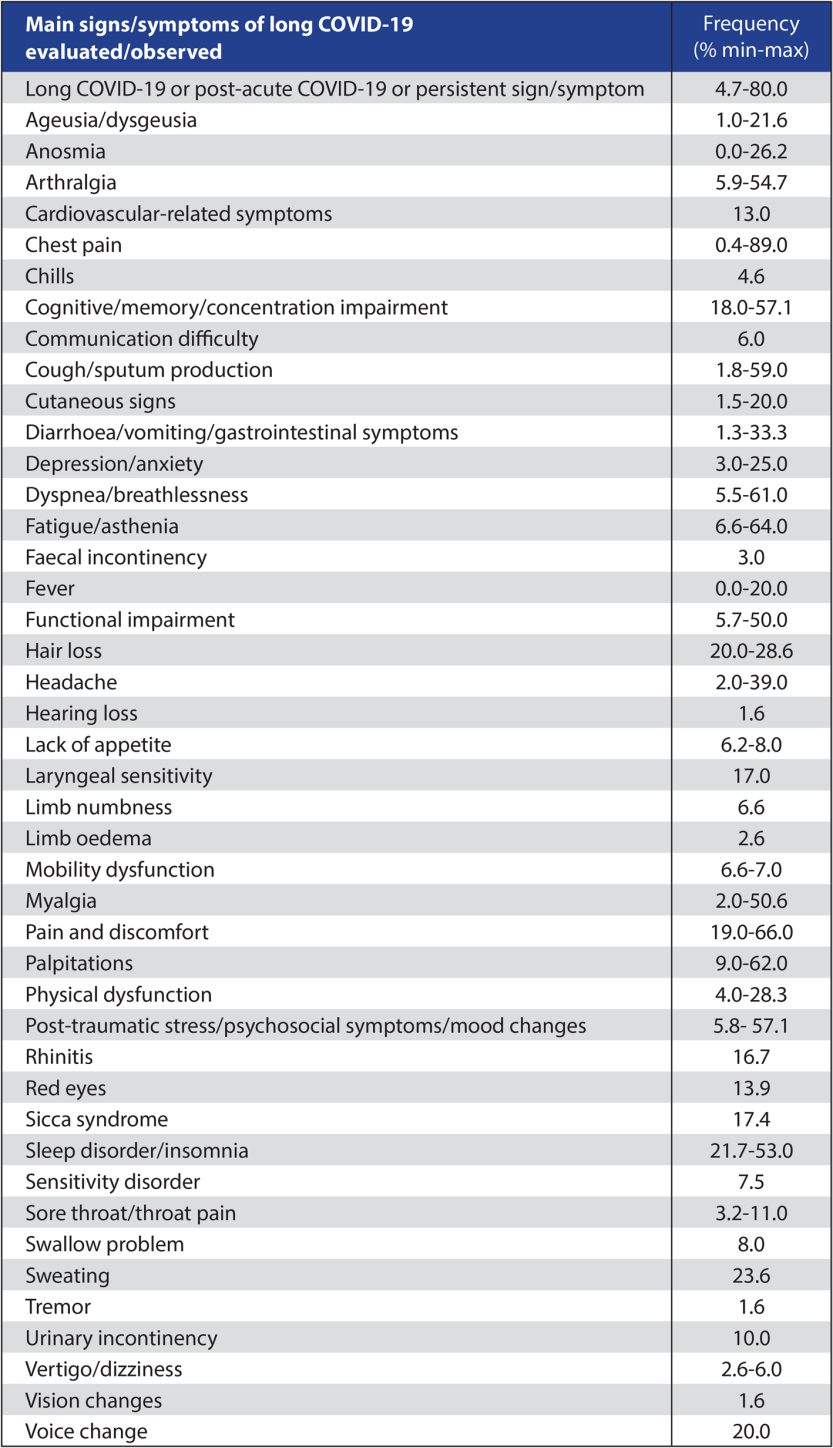
Symptoms and frequency of occurrence (% of LC cases) (with permission of Cabrera Martimbianco et al. (2021))

The current body of evidence opens new pathways for unraveling the mystery of LC. In this context, the “nicotine hypothesis” presented by Changeux et al. (2020) is of groundbreaking importance (Changeux 2020). The authors point to a proline-arginine-arginine-alanine (PRRA) amino acid sequence (AAS) in the head region of the spike glycoprotein (SGP) of SARS-CoV-2, located at the interface between the S1 and S2 domains, just upstream of the S1/S2 cleavage site (Walls et al. 2020). This AAS in the Y674-R685 region of SARS-CoV-2 is similar to that of cholinergic toxins (e.g., from snakes of the genera Ophiophagus [cobra] and Bungarus, the G-ectodomains of three rabies lyssavirus strains [formerly rabies virus RABV] (Changeux 2020) or the muscarinic toxin-like protein and cobratoxin [naja siamensis] (Farsalinos 2020), whose main action is to block nicotinic acetylcholine receptors (nAChRs). The remarkably high AAS concordance led the authors to propose that SARS-CoV-2 functions as an nAChR antagonist, likely due to its potential binding affinity for these receptors (Changeux 2020). This assumption was later confirmed in further in silico and in vitro studies (Farsalinos 2020); Carlson 2023). Since, unexceptional every cell, even on the subcellular level (e.g. mitochondria), is subject to cholinergic neurotransmission in terms of its metabolic and functional regulation (Proceedings of the 2nd International Symposium on Non-Neuronal Acetylcholine 2007), a new pathogenetic understanding for these post-viral symptom complexes is possible. The alkaloid nicotine, with its extraordinarily high affinity for nAChRs, could enable competitive displacement of the virus particles to initiate immunological processing by preformed antibodies. This resulted in numerous instances of self-medication using nicotine patches (low-dose transcutaneous nicotine – LDTN) by LC/ME/CFS patients, with impressive improvements in many of the cases and insights into the LC/ME/CFS root causes to provide clearer subtyping (see Supplement 1). This review integrates current knowledge to connect pathological findings in LC with impaired cellular cholinergic neurotransmission and proposes conclusions for a therapeutic approach (Leitzke 2023).

Furthermore, in an initial case study, we demonstrate alterations in vivo in nAChR imaging using simultaneous whole-body PET/CT and PET/MRI, along with significant clinical improvement following LDTN. This supports the underlying pathophysiological hypothesis and validates the proposed approach.

### Acetylcholine receptors

Acetylcholine receptors (AChRs) are classified into ionotropic nicotinic (nAChRs) (Gotti et al. 2006) and metabotropic muscarinic (mAChRs) (Jones 1992) receptors. These two groups are distinguishable by different binding behavior to nicotine and muscarine and the mode of signal transmission (Jones 1992; Graef et al. 2011). MAChRs usually act slowly by coupling to G-proteins that activate phospholipase C (PLC) or inhibit adenylate cyclase (Scarr 2012). In addition, non-canonical signaling pathways involving phospholipase A2, phospholipase D, tyrosine kinase, and calcium channels are modulated by mAChRs (Scarr 2012). Whether mAChRs act in an excitatory or inhibitory manner depends on the type of cell they target (Scarr 2012). Neuronal, non-neuronal and muscle-type nAChRs consist of a pentameric composition of nine α (α2-α10), three β (β2-β4) and γ, ϵ or δ subunits (Osipov et al. 2023), which assemble in a homomeric (α7) or heteromeric combination to fast-acting, excitatory membrane-bound channels allowing rapid cellular influx of cations upon activation (Gotti et al. 2006). Thus, activation of nAChRs in the brain leads to increased release of several important neurotransmitters, including dopamine, serotonin, glutamate, and gamma-aminobutyric acid (GABA) (Taly et al. 2009). The allosteric configuration of nAChRs is crucial for their function. Together with the orthosteric binding sites (OBS) for acetylcholine (ACh), there are allosteric binding sites (ABS) at the interface between α- and non-α-subunits. ABS can bind to a variety of transmitters or proteins that modulate the molecular quaternary structure of the receptor to increase (positive allosteric modulators [PAMs]) or decrease (negative allosteric modulators [NAMs]) the overall conductance of the channel by altering the steric energy required for a further conformational change (Kenakin 2024). The excitability of each cell is therefore subject to a variety of external influences, at the center of which is the coupling of ACh to its OBS (Spurny 2015).

### Neuropathology in long COVID

**Fig. 1:**
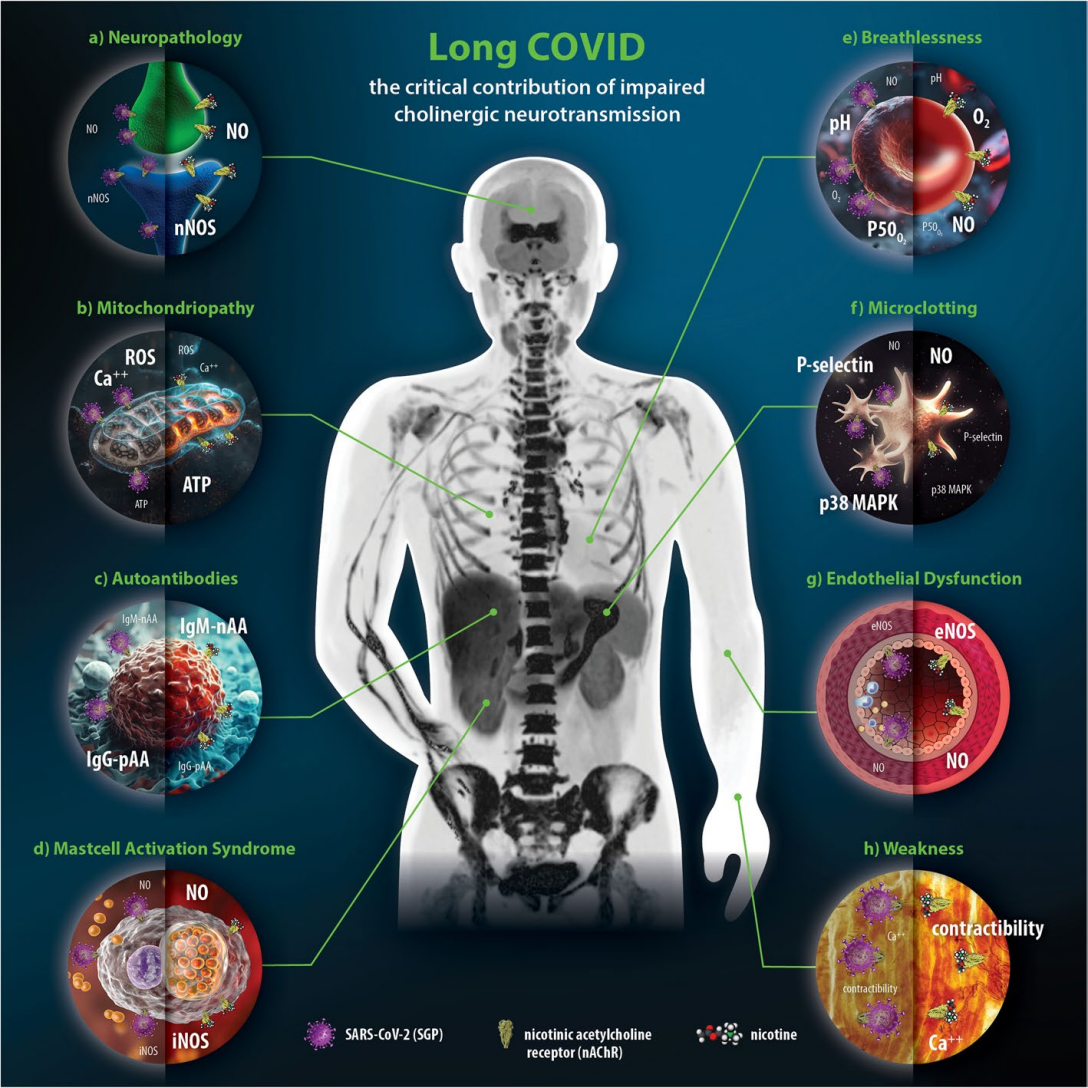
The consequences of spike glycoprotein (SGP)-related blockade of nicotinic acetylcholine receptors (nAChRs) are shown based on the individual organ systems affected, with receptor blockade and its consequences shown in the left semicircle and normal cholinergic receptor function after SGP displacement by nicotine shown in the right semicircle. **a** impaired cholinergic neuromodulation with reduced nitric oxide (NO) and impaired neuronal excitability; **b** impaired mitochondrial acetylcholine action with reduced adenosine triphosphate (ATP) synthesis capacity and reduced reactive oxygen species (ROS) and Ca^++^ clearance; **c** impaired b-lymphocytic cholinergic innervation with natural autoantibodies (nAA) to pathological autoantibodies (pAA) shift; **d** impaired mastocytic cholinergic innervation with reduced inducible NO synthetase (iNOS) and inadequate mast cell degranulation; **e** impaired acetylcholine action on erythrocytes with decreased erythrocyte elasticity and oxygen uptake with increased pH/P50_O2_ and rightward shift of oxygen dissociation curve (ODC); **f** impaired thrombocyte cholinergic innervation with increased P-selectin expression and increased aggregability **g** impaired endothelial cholinergic innervation with reduced endothelial NO synthetase (eNOS) synthesis, vasoconstriction, leukocyte adhesion and thrombogenesis; **h** SGP-induced blockade of neuromuscular junctions leads to reduced strength and painful overloading of unblocked muscle fibrils

Patients with LC suffer from cognitive dysfunction (Ceban 2022), poor concentration (López-Hernández 2023), memory loss (López-Hernández 2023), fatigue (López-Hernández 2023), depression (Ceban 2022), and other neurological disorders (Stefanou 2022). The so-called "brain fog" is characterized by an inability to think clearly (Kavanagh 2022). In discussing the associated damage patterns, Leng et al. (2023) suggest direct viral neuroinvasion (Leng 2023). Other authors consider inflammatory mechanisms (Kavanagh 2022; Davis et al. 2023) perpetuated by cytokines (Kavanagh 2022; Davis et al. 2023; Monje and Iwasaki 2022), chemokines, and migrating immune cells (Davis et al. 2023) to be likely. Neuronal apoptosis (Iadecola et al. 2020) or hypoxia (Rutkai 2022) as well as circulatory central nervous system (CNS) dysfunction (Aj 2023) and neurotransmitter imbalances (Monje and Iwasaki 2022), microglial activation (Leng 2023) or impairment of certain receptor groups (Reiken et al. 2022) have also been associated with LC-associated neuropathy. All neurological symptoms of LC share low specificity (López-Hernández 2023) and highly fluctuating occurrence (O'Brien 2023), which suggests that these disorders may have a functional origin. We recently highlighted the neuromodulatory function of nAChRs (Leitzke 2023). Pre- and postsynaptic and axonal nAChRs regulate the amount and rate of transmitter release at all central and peripheral synapses (Gotti et al. 2006; Picciotto et al. 2012) and ensure a coordinated and adaptive interaction of all neuronal signals and adaptation of signaling to the situational requirements of the entire organism (Leitzke 2023; Picciotto et al. 2012). It also controls the excitability and responsiveness of axons and central and autonomic nuclei to stimulation in general (Picciotto et al. 2012). The brain-specific neuronal NO synthase (nNOS) is involved in modulation of long-term synaptic transmission and thus learning, memory, and neurogenesis (Zhou and Zhu 2009). Its obligate Ca^++^dependence (Cyr et al. 2020) suggests a close functional relationship with nAChRs as Ca^++^channels. Graef et al. (2011) found in their review that nAChRs are involved in the regulation of cognition, positioning them as key regulators of attention and working memory (Graef et al. 2011) (Fig. 1a). Viral blockade of neuronal AChRs with marked impairment of cholinergic neuromodulation offers a very plausible etiopathogenetic explanation for LC-associated neuropathology (Leitzke 2023).

### Mitochondriopathy in long COVID

Numerous authors have suggested that mitochondriopathy is the central pathologic element of LC. However, neither conclusive data nor etiopathogenetic models are available (Sakellaropoulos et al. 2022). Nevertheless, there are LC/ME/CFS-specific clinical findings such as post-exertional malaise (PEM), fatigue and loss of strength that suggest a general cellular energy deficiency (Sakellaropoulos et al. 2022). Mitochondria are the central energy suppliers of the cell, providing adenosine triphosphate (ATP) via oxidative phosphorylation (OXPHOS) (Mali 2019). Metabolically, mitochondria are responsible for β-oxidation of fatty acids (Friedman 2014), steroid synthesis, Fe-S center formation, and heme synthesis (Friedman 2014). They are also the central structure for cytosolic Ca^++ ^buffering, thermogenesis, and the initiation of apoptosis (Friedman 2014). In addition, the electron transport chain (ETC) is the site of origin for reactive oxygen species (ROS) (Friedman 2014). The impairment of these core processes of cellular metabolism is characterized by decreased ATP synthesis, inefficient Ca^++ ^buffering and increased ROS production. In addition, pro-apoptotic factors are released (Mali 2019). Several nAChR subtypes, including α7, α3β2, α4β2, α7β2, and α9, have been identified on the outer mitochondrial membrane (OMM) in isolated mitochondria (Lykhmus et al. 2014). The distribution of the different subtypes is tissue specific (Friedman 2014; Skok 2022). They are responsible for the activation of intramitochondrial kinases such as Ca-calmodulin-activated kinase II (CaKMII), Src kinases, and phosphatidylinositol 3-kinase (PI3K) (Skok 2022). An important function of kinases is the post-transcriptional modification (PTM) of functional proteins. This, regarding mitochondria, means that activated kinases jointly orchestrate mitochondrial signaling and metabolic pathways thus providing reliable protection against pro-apoptotic factors such as Ca^++^and ROS accumulation, and ensuring a continuous cellular supply of ATP (Skok 2022) (Fig. 1b). In their review, Chang et al. (2021) linked the endothelial dysfunction observed in COVID-19 to increased oxidative stress caused by mitochondrial ROS overexpression (Chang 2021).

The associations described suggest that viral blockade of mitochondrial nAChRs is most likely an important component of LC.

### Autoantibodies in long COVID

Natural autoantibodies (nAAs) are encoded by non-mutated V(D)J gene sequences (Schettino 1997) and are synthesized in CD5^+^cells (Casali 1987) of the neonatal B cell reservoir (Ma 2022). They are part of the physiological immune system, albeit in small quantities. These mostly IgM-nAAs (Ma 2022) are an important component of immunological homeostasis (Wildbaum et al. 2003) and the first line of defense (Zhou et al. 2007). However, they have no autoaggressive potential (Ma 2022). Pathological, auto-aggressive, somatically mutated IgG-type autoantibodies (pAAs) (Ma 2022) can develop under the influence of toxins, chemicals, viral infections and excessive T-cell stimulation (Elkon and Casali 2008). These high-affinity monoreactive IgG-pAA are objective markers of pathoimmunologic processes and are associated with disturbed cellular homeostasis, altered antigen receptor signaling pathways and cell effector functions (Elkon and Casali 2008). The virus-induced switch from nAA to pAA (Elkon and Casali 2008) is attributed to molecular mimicry (Zhao 1998), with nAA acting as a template for the synthesis of pAA (Fleming 2006). Several authors report the observation of such a switch with increased pAA synthesis in the context of acute SARS-CoV-2 infection (Chang 2021; Damoiseaux 2022). In addition, there is evidence that pAAs cause a variety of LC symptoms (Ortona and Malorni 2022) via increased platelet clotting (Zuo 2020) and vascular or tissue inflammation (Cochrane 1973). Immune cells, including dendritic cells, T and B lymphocytes, monocytes and macrophages, express a considerable number of different AChRs (Fujii 2017). These include nAChRs with the subunits α2, α5, α6 (Fujii 2017; Kawashima et al. 2007), α7, α9, and α10 (Fujii 2017; Plazas 2005) as well as α4, ß2, and ß4 (Plazas 2005) and all known mAChRs (M1-M5) (Fujii 2017; Kawashima et al. 2007). Other components of the cholinergic system such as choline acetyltransferase (ChAT) and ACh (Fujii 2017) are also expressed by T and B lymphocytes. They regulate the production of proinflammatory cytokines such as tumor necrosis factor α (TNFα) and interleukin 2 (IL2) (Fujii 2017) through α7nAChRs via the so-called cholinergic anti-inflammatory pathway—CAP (Tracey 2007). This suggests a partial autocrine/paracrine regulation of the immunologic activation of both innate and humoral immune responses (Fujii 2017) and is particularly relevant because TNFα, IL6 and interferon-γ (IFNγ), which are overexpressed in spleen cells of α7 KO mice (Fujii 2007), play a major role in the nAA-IgM to pAA-IgG switch (Fujii 2017) (Fig. 1c). Rodent B lymphocytes mainly express homomeric α7 and heteromeric α4β2 receptors (Skok et al. 2005). Epibatidin binding sites, representing heteromeric receptors (Sihver et al. 1999), and bungarotoxin binding sites, specific for homomeric receptors (McLane 1991), were found in large numbers (12,200 ± 3200 per cell and 3130 ± 750 per cell, respectively) (Skok et al. 2005). AChRs are key regulators of antibody synthesis (Skok 2003). M1/M5 KO mice had significantly lower levels of antigen-specific IgG1 and serum cytokines compared to WT mice (Fujii 2007). In contrast, α7-KO mice had significantly increased levels of ovalbumin (OVA)-specific IgG1 two weeks after OVA immunization compared to WT C57BL/6 J mice (Fujii 2007). B-cell proliferation after stimulation with anti-CD40 or anti-B-cell receptor antibodies was also significantly greater in α7-KO mice or in α7nAChRs inhibited with methyllycaconitine (MLA) than in WT mice (Koval 2011; Skok et al. 2007). In conclusion, mAChRs upregulate both B lymphocyte numbers and antibody production, whereas nAChRs have an inhibitory effect on B cell function and proliferation (Fujii 2007; Fujii et al. 2003). In this context, it is noteworthy that the administration of nicotine resulted in a 30% reduction in the CD40-induced proliferation of β2 KO cells (Skok et al. 2005). In a retrospective analysis, Peng et al. (2023) compared data from previous PCR-confirmed COVID-19 infections (*n* = 1,028,721) with COVID-19-negative controls (*n*= 3,168,467) with regard to the risk of autoimmune diseases (AD) (Peng 2023). There was a significantly higher risk of developing AD (pernicious anemia, spondyloarthritis, rheumatoid arthritis, other autoimmune arthritis, pemphigoid, Graves' disease, antiphospholipid antibody syndrome, immune-mediated thrombocytopenia, multiple sclerosis, and vasculitis) in COVID-19-positive individuals (Peng 2023) (Table 2).

**Table 2:**
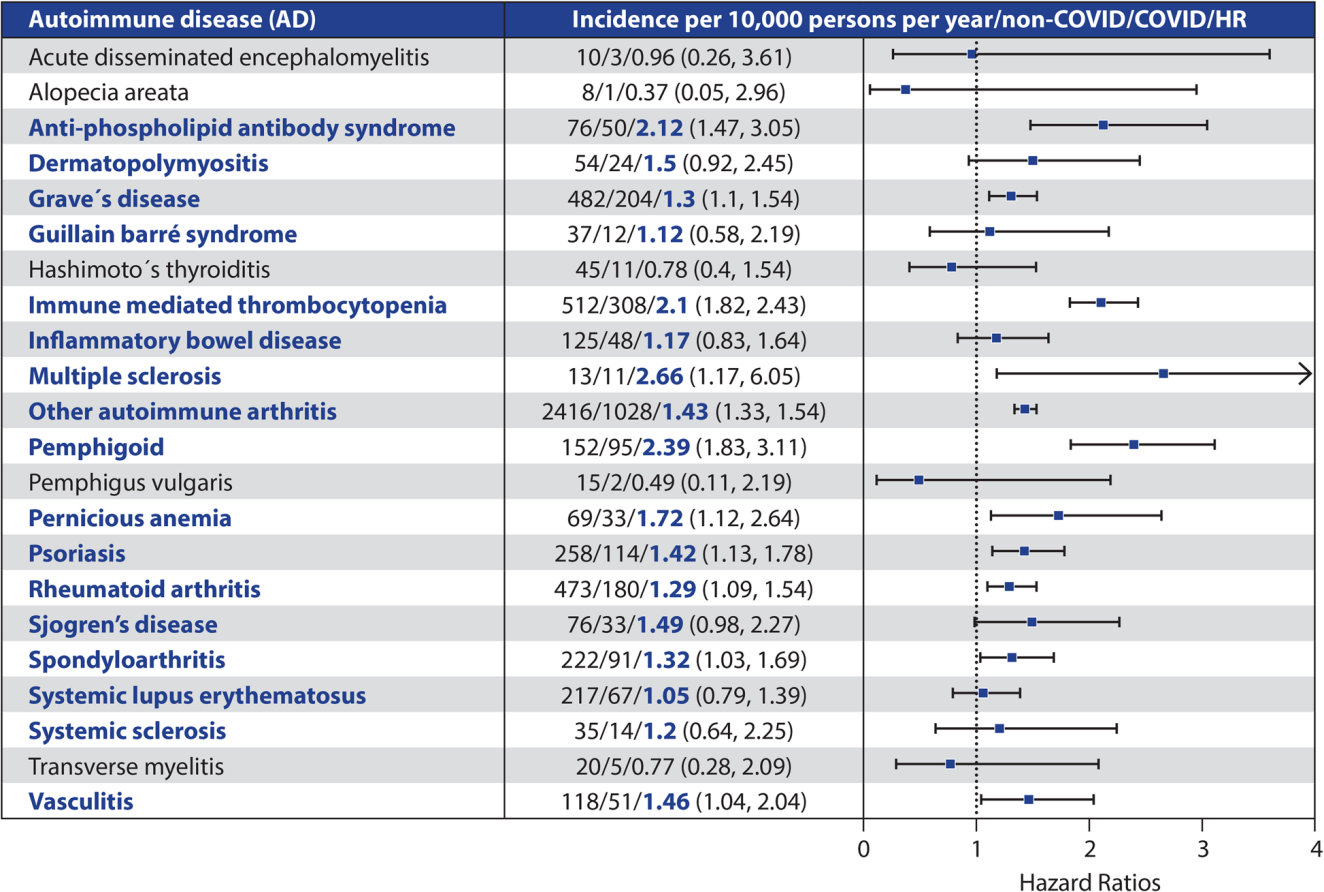
Impact of COVID-19 on the risk of newly diagnosed autoimmune disease (COVID-19 versus non-COVID-19). Hazard ratio (HR) and 95% confidence interval (95% CI) estimated by Cox regression. HR > 1 (or <1) means that patients with COVID-19 had a higher (lower) risk of newly diagnosed autoimmune disease than the control cohort without COVID-19. Error bars indicate the 95% CI of the respective HR (with permission of Peng et al. (2023))

The described correlations explain the high incidence of IgG-type pAA associated with COVID-19 due to SARS-CoV-2 induced T-cell stimulation leading to a shift from nAA-IgM to pAA-IgG. Blockade of membrane-bound nAChRs of B cells by SGP may perpetuate this effect through release of proinflammatory cytokines with further nAA-IgM/pAA-IgG shift. The persistent disruption of nAChR-driven inhibition of inappropriate B cell proliferation and antibody overproduction would be a plausible cause of auto-aggressive immune dysregulation with subsequent ADs in LC.

### Mast cell activation syndrome (MCAS) in long COVID

As a central component of the innate immune system, mast cells (MCs) are the first line of defense against external and internal threats. This includes allergic responses (Vitte et al. 2022) as well as defense against microbial threats, maintenance of tissue homeostasis, and modulation of the adaptive immune system (Xu 2020). MCs are particularly abundant on surfaces in contact with the external environment (Galli et al. 2011) such as the mucous membranes of the digestive and respiratory tracts, the skin, and the meninges of the brain (Dudeck 2019). The close anatomical relationship between neuronal structures and MCs illustrates their mutual control (Godinho-Silva 2019). For example, the MC membrane contains numerous receptors for classical neurotransmitters such as ACh, NO, gamma-aminobutyric acid (GABA), dopamine (DA), and glutamate (Xu 2020).

In addition, there are neuropeptide receptors for substance P (SP), vasoactive intestinal peptide (VIP), corticotropin-releasing factor (CRF), calcitonin gene-related peptide (CGRP), neurotrophins (NTs) and neurotensin (NT), adenosine triphosphate (ATP), tachykinin, opioid peptides, and others (Xu 2020). Among the MC AChRs (Mishra 2010), the α7nAChRs are the most important (Xu 2020), explaining their central role in limiting the release of proinflammatory cytokines via the CAP (Tracey 2007). Stimulation of these receptors reduces MC degranulation (Fig. 1d). Thus, administration of α7nAChR agonists (e.g., GTS-21) was followed by inhibition of IgE-induced MC degranulation in mouse bone marrow-derived MCs (Kageyama-Yahara et al. 2008). TNFα release from MCs reduced after lipopolysaccharide (LPS) challenge (Guzmán-Mejía et al. 2018) and a reduction in interleukin-6 (IL-6) release and NO efflux from mononuclear cells in chronic obstructive pulmonary disease (COPD) have also been demonstrated with α7nAChR agonists (Douaoui 2020). Endogenous NO synthesis is catalyzed by three enzymes (neuronal NO synthase — nNOS, inducible NO synthase — iNOS, and endothelial NO synthase — eNOS). All these NO forms, as well as exogenous NO donors (e.g., sodium nitroprusside [SNP]) (Xu 2020) result in mastocyte degranulation inhibition. Other NO donors (spermine-NO and SIN-1) showed an inhibitory effect on IgE-induced MC degranulation in peritoneal and mesenteric MCs, but also a reduction in the expression of mRNA for IL4, IL6 and TNFα in rat MCs (Coleman 2002; Davis 2004). Furthermore, this effect could be reversed with NOS inhibitors such as NG-nitro-L-arginine methyl ester (L-NAME) (Masini et al. 1991). At the molecular level, the effect of NO can be attributed to inhibition of the cysteine protease calpain. Calpain is associated with cellular integrin activation, has an adhesion-promoting effect on MCs, and is inhibited by NO via nitrosylation of a thiol group at its active site (Forsythe 2003). Furthermore, there is a pronounced bidirectional dependence between the effect of NO and nAChRs (Xu 2020) (described in detail in the "Endothelial Dysfunction" section). In mast cell activation syndrome (MCAS), nonspecific triggers (food, odors, stress, exercise, drugs, or temperature changes) (Arun 2022) lead to inappropriate mast cell degranulation with the release of histamine, heparin, cytokines, prostaglandins, leukotrienes, and proteases. This affects a large number of LC patients (Weinstock et al. 2021; Afrin et al. 2020). Therefore, some authors suggest that MCs play a central role in LC pathology (Weinstock et al. 2021; Afrin et al. 2020). Weinstock et al. (2021) investigated the prevalence of MCAS in LC patients and compared 136 LC patients with 80 true MCAS patients and 136 healthy volunteers (Weinstock et al. 2021). They found almost identical Mast Cell Mediated Release Syndrome (MCMRS) scores in LC patients compared to patients with primary, untreated MCAS (Weinstock et al. 2021). The interpretation of MCAS in LC based on blockade of one of the key inhibitory regulators of MC degranulation (i.e. nAChRs) by SGP seems to be a very likely scenario.

### Dyspnea and hypothermic, pale, and painful extremities

Patients with LC often complain of recurrent dyspnea (López-Hernández 2023) and painful, acrocyanotic extremities (Iftekhar and Sivan 2023). However, these complaints usually have no spirometric or imaging pathologic correlate (Frizzelli 2022) and the subjective dyspnea does not appear to have a pulmonary cause. The extremely high erythrocyte concentration of the membrane-spanning enzyme acetylcholinesterase (AChE) illustrates the central functional importance of ACh for these cells (Saldanha 2017). Membrane-bound erythrocyte nAChRs regulate the deformability and oxygen-binding capacity of red blood cells (RBCs) (Almeida et al. 2008).

Their activation increases erythrocyte Ca^++^concentration and deformability in an NO-dependent manner (De Almeida and Saldanha 2010), while decreasing oxygen P50, erythrocyte plasma pH, Na^+^ and K^+^concentrations (Mesquita et al. 2001) and erythrocyte aggregation propensity (Mesquita et al. 2001). Thus, ACh is at the center of physiological requirements for pulmonary and tissue gas exchange, both in terms of blood viscosity and erythrocyte oxygen-carrying capacity. Perturbations in erythrocyte cholinergic signaling result in NO efflux from the cell (Mesquita 2000) and, due to the increase in intraerythrocytic pH (De Almeida and Saldanha 2010) and oxygen P50, a rightward shift of the hemoglobin oxygen dissociation curve (ODC) (Saldanha 2018) (Fig. 1e). The increased RBC stiffness associated with cholinergic under-representation is caused by a decrease in membrane lipid fluidity (Almeida et al. 2008). This leads to an increase in vascular shear stress (De Almeida and Saldanha 2010) and impassability of erythrocytes in microvessels, which are smaller in diameter than erythrocytes (Almeida et al. 2008). For pulmonary gas exchange, this means that arterial oxygen content is reduced both by a lower oxygen binding capacity of hemoglobin and by impaired pulmonary capillary passage of erythrocytes due to cell stiffness (Saldanha 2018).

The increased respiratory drive and breathlessness, which inversely correlate with arterial oxygen partial pressure (paO_2_) (Pflüger 1868), are likely caused by the P50 increase and the rightward shift of the ODC due to reduced cholinergic innervation of erythrocytes (De Almeida and Saldanha 2010). The problem of tissue hypoxia is aggravated by the increased tendency of erythrocytes to aggregate (Mesquita et al. 2001), also due to cholinergic under-representation. Thus, the increased serum lactate levels in LC described by several authors (Ghali 2019; Lane 1998) might be due to the increased anaerobic metabolism caused by oxygen deprivation. In addition, Doyle and Walker (1990) showed that pulmonary arterial pressure increases during perfusion of the pulmonary circulation with less elastic erythrocytes (Doyle 1990), which is another known cause of dyspnea (Blasi 2012).

In summary, both the intermittent dyspnea reported by patients and the occurrence of hypothermic, pale, and painful extremities could be explained by the viral impairment of the acetylcholine-dependent mechanisms on erythrocytes described above.

### Clotting dysfunction in long COVID

Serologic hyper-coagulopathies associated with a thrombocytopenic constellation have been observed in severe COVID-19 (Leitzke and Schönknecht 2021; Tang et al. 2020), with thrombocytopenia partially attributed to SARS-CoV-2-associated thrombopoietin (TPO)-neutralizing pAAs (Nunez-Castilla 2021). In contrast, the prothrombotic phenomena associated with LC are mainly related to platelet-derived cellular mechanisms and are referred to as "microclotting" (Kell 2023). P-selectin (PS), a glycoprotein from the C-type lectin family, is rapidly released upon cellular activation from the α-granules of platelets and the Weibel-Palade bodies of endothelial cells (Hawiger 1995; Merten 2000). At the cell surface, PS mediates platelet and neutrophil rolling on activated endothelial cells (Frenette 2000) and controls neutrophil-monocyte-platelet interactions (Larsen 1989). After GP IIb/IIIa binds to soluble fibrinogen to bridge adjacent platelets or platelets and endothelial cells, PS stabilizes the initiated aggregation (Hawiger 1995). Thus, PS is not only the major determinant of the size and stability of platelet aggregates (Merten 2000) but also has a platelet-activating effect (Jankauskaite et al. 2022) and leads to a procoagulant shift in the prothrombotic-fibrinolytic balance by stimulating monocyte tissue factor (TF) (Frantzeskaki et al. 2017). Pretorius et al. (2022) found a moderate degree of platelet activation (mean severity of 7.1 out of 10) due to significantly elevated PS (CD62P) levels in platelet-poor plasma (PPP) in 30 of 80 LC patients studied (Pretorius 2022). α7nAChRs expressed on the platelet membrane control platelet activation (Bennett 2018) by inhibiting p38 mitogen-activated protein kinase (MAPK), which is critical for platelet activation and aggregation, thrombus formation, and consolidation (Li et al. 2001) and is one of the earliest intracellular platelet-activating responses to a variety of stimuli, including mitogens, cytokines, stress, and ROS (Shi 2017; Son 2013). ACh is a potent inhibitor of p38 MAPK. It prevents phosphorylation and thus activation of p38 MAPK (Zhao 2015) (Fig. 1f). Bennett et al. (2018) showed that agonistic stimulation of platelet nAChRs leads to an increase in intracellular nitric oxide (NO) levels, resulting in inhibition of both P-selectin release and GPIIb/IIIa activation (Bennett 2018). In addition to AChRs, central components of the cholinergic system such as ACh, ChAT, AChE have been detected in platelets (Wessler and Kirkpatrick 2008) suggesting an autocrine involvement in platelet activation (Schedel et al. 2011) and, via α7nAChRs in the megakaryocytic cell line, also an autocrine inhibition of thrombopoiesis (Schedel et al. 2011). Bennett et al. (2018) showed in their ex vivo study that an nAChR antagonist (pancuronium) had prothrombotic effects, while an AChE inhibitor (donepezil) inhibited platelet activation. These observations suggest that platelet-released ACh acts as an endogenous inhibitor of platelet activation (Bennett 2018). In their review, Jankauskaite et al. (2022) again found that thrombocytic nAChRs orchestrate the inhibition of PS expression (Jankauskaite et al. 2022). It is noteworthy that P-selectin overexpression cannot be reversed by administration of GP IIb/IIIa inhibitors, acetylsalicylic acid, or oral anticoagulants that are not vitamin K antagonists (NOACs) (Merten 2000). These correlations make a causal involvement of blocked nAChRs in LC-associated perfusion disorders in the sense of microclotting very likely.

### Endothelial dysfunction in long COVID

Oikonomou et al. (2022) studied 73 COVID-19 patients using flow-mediated dilation (FMD), a validated test to assess systemic endothelial dysfunction (Oikonomou 2022). FMD studies in Sprang-Dawley rats with phenylephrine-constricted carotid arteries showed a significant improvement in FMD and endothelial NO concentrations with ACh administration as visualized by diaminofluorescein-FM diacetate (DAF-FM) (Wilson 2016). In the studies by Oikonomou et al. 2022 endothelial function was significantly impaired during acute COVID-19 infection (1.65 ± 2.31% vs. 6.51 ± 2.91%). The measured values were inversely correlated with interleukin-6 (IL-6) and troponin levels (as markers of disease severity), and this impairment did not fully recover despite an overall improvement over 1 and 6 months of follow-up compared to controls (acute: 1.75 ± 2.19% vs. 1 month: 4.23 ± 2.02%, vs. 6 months: 5.24 ± 1.62%;*p*= 0.001) (Oikonomou 2022). Also using FMD, Ambrosino et al. (2021) showed in 133 convalescent COVID-19 patients with persistent, predominantly pulmonary, impairment that two months after a negative SARS-CoV-2 swab there was a significant impairment in systemic endothelial function compared to controls (3.2% ± 2.6 vs. 6.4% ± 4.1*p*< 0.001) (Ambrosino 2021). As the most extensive organ in the body, the endothelium is a central regulator of hemostasis, vascular resistance, permeability, innate immunity, barrier function, oxidative stress, and functional integrity of perfused tissues (Xu 2021). Numerous endothelial vasoactive substances control the balance of vasoconstriction and vasodilation, proliferation and inhibition of proliferation, prothrombotic and antithrombotic, inflammatory and anti-inflammatory, fibrinolytic and antifibrinolytic, and oxidative and antioxidative processes (Xu 2021). NO, produced by Ca^++^-activated (Corretti 2002) endothelial NO synthase (eNOS), is the major vasodilator molecule that also promotes antithrombotic, fibrinolytic, anti-inflammatory, antiproliferative, and antioxidant endothelial effects (Cyr et al. 2020; Xu 2021). Its antithrombotic effect is achieved by inhibiting the adhesion of leukocytes and platelets (Cyr et al. 2020) to activated endothelial cells (ECs), thereby suppressing the formation of inflammation-related thrombi or atherosclerotic plaques (Xu 2021). The vasodilator and antithrombotic NO effects are counteracted by several vasoconstrictors. These include angiotensin II (AT2), endothelin I (ET1), and reactive oxygen species (ROS), which in turn directly reduce NO by inactivating and uncoupling eNOS (Cyr et al. 2020). After endothelial synthesis, NO is released from ECs into vascular smooth muscle, where it causes relaxation (Furchgott and Vanhoutte 1989). Intravascularly, erythrocytes serve as NO reservoirs (Furchgott and Vanhoutte 1989). The band 3 protein (B3P) serves as a pathway for bidirectional passage of NO from and into erythrocytes (Huang 2001) where NO storage occurs as S-nitrosohemoglobin (SNOHb) (Huang 2001). When needed, erythrocytes release NO from SNOHb binding by transferring NO to the thiol group of B3P, thereby expelling it into the vascular lumen (Pawloski 2005). These mechanisms are characterized by high efficiency and capacity and are essential for the functional integrity of endothelial function. As a result, the definition of ED as a reduced bioavailability of NO and thus a shift in the balance between vasoconstrictor and vasodilator as well as prothrombotic and antithrombotic effects in favor of vasoconstrictor and prothrombotic effects (Cyr et al. 2020) is well established. In primary cell cultures, nAChRs with α3, α5, β2, and β4 subunits have been detected on the membranes of human aortic ECs (Macklin et al. 1998) and α2, α3, α4, α5, α7, and β2 and β4 substituted nAChRs on rat microvascular ECs (Moccia 2004). In ECs, stimulation of membrane-bound nAChRs increases intracellular NO levels (Kirkpatrick et al. 2003), which in turn is responsible for vascular relaxation (Furchgott and Zawadzki 1980). This effect of nAChRs results from the Ca^++^dependence of eNOS activity (Corretti 2002) and the primary function of nAChRs as Ca^++^channels (Kalamida 2007). Accordingly, viral blockade of endothelial nAChRs leads to a reduction in NO and a shift in the balance of vasomotor and coagulation toward vasoconstriction and thrombogenesis (Cyr et al. 2020; Saldanha 2018; Furchgott and Zawadzki 1980) (Fig. 1g). This is followed by reduced blood flow and tissue hypoxia, as well as vascular shear stress, which in turn contributes to damage to the glycocalyx (GC) (Bai and Wang 2014) (the gel-like inner vascular layer of proteoglycans and glycosaminoglycans (Furchgott and Zawadzki 1980) which subsequently protectively modulates leukocyte-endothelial interactions, thrombus formation, and vascular permeability (Wu et al. 2024), with further enhancement of ED (Wu et al. 2024). Vollenberg et al. (2021) demonstrated elevated syndecan-1 levels 88 days after mild SARS-CoV-2 infection, which are specific for persistent GC damage (Vollenberg 2021). This increase was also observed 4 and 12 months after COVID-19 infection (Lambadiari 2021; Ikonomidis 2022). The correlations described above indicate that the pathological endothelial processes observed in LC are possibly due to an initial blockade of endothelial nAChRs by the SARS-CoV-2 virus.

### Arthralgia/myalgia

Despite various diagnostic approaches such as bioelectrical impedance analysis (BIA), computed tomography (CT) and ultrasound (US), no structural or pathogenetic correlate has been found for the muscle pain and weakness that occurs in up to 66.7% (López-Hernández 2023) of LC cases (Montes-Ibarra et al. 2022). It is therefore suggested that persistent inflammation with impaired muscle protein metabolism, loss of physical activity and inadequate nutritional intake are the likely causes of muscle dysfunction associated with LC (Montes-Ibarra et al. 2022). Guy-Coichard et al. (2008) investigated the disease-related pain burden in 511 patients with neuromuscular diseases (NMD). They found that 67.3% of NMD patients reported a significant pain burden and that myasthenia gravis (MG) and metabolic myopathy had the highest pain intensity (severe, partially immobilizing pain) at 38% and 49%, respectively (Guy-Coichard 2008). The example of MG shows that the quantitative reduction of functional neuromuscular junctions (NMJ) is an independent risk factor for muscle pain, which could result from an overload of the remaining physiologically innervated muscle fibrils (Fig. 1h). Indeed, many authors have reported SARS-CoV-2-induced myasthenia gravis (Essajee et al. 2021; Karimi et al. 2021). Striated muscle nAChRs, essential for the function of the NMJ (Di 2013), and the pain-modulating role of neuronal nAChRs (Posselt 1829) offer a plausible explanation for both muscle weakness and pain caused by SGP-related nAChR blockade. The results of Peng et al. (2023) show that an autoimmune genesis of joint and muscle pain in LC (Peng 2023) is also conceivable, which can also be attributed to nAChR blockade of B cells (see section "Autoantibodies in LC").

## Postural orthostatic tachycardia syndrome (POTS)

During selective perfusion of intracardiac ganglia with α-conotoxin MII, a specific antagonist of the α3/β2 interface of nAChRs, Bibevski et al. (2000) showed a reversible attenuation of the cardiac sinus cycle length (SCL) response to preganglionic vagal stimulation by approximately 70%. Local administration of α-conotoxin AuIB, a specific antagonist of the α3/β4 interface, and α-bungarotoxin, a specific α7nAChR blocker, attenuated this vagal mechanism by approximately 20% and 30%, respectively (Bibevski 2000). The authors concluded that despite the extensive muscarinic cholinergic innervation of the heart (Osipov et al. 2023), α3/β2 nAChRs (α3/β4 and α7nAChRs to a lesser extent) play an important role in vagal control of heart rate (Bibevski 2000). Since negative chronotropic control of the heart during orthostasis relies on rapid vagal signaling, it is more plausible that orthostatic rate control is governed by fast-acting ionotropic nicotinic AChRs rather than slower-acting metabotropic mAChRs.

The hypothesized viral blockade of intracardiac ganglionic nAChRs provides a rational explanation for the previously unexplained phenomenon of postural orthostatic tachycardia syndrome (POTS), which is frequently observed in LC (Abbate 2023).

### Postvaccination syndrome

The ongoing controversy over the existence of PVS is being replaced by an increasing number of reports describing an LC-like spectrum of symptoms following SARS-CoV-2 vaccination (Krumholz 2023; Abicic et al. 2022). As with LC, the pathophysiological background of this phenomenon remains unclear (Turner et al. 2023). Symptoms of PVS include exercise intolerance, excessive fatigue, numbness, brain fog, and neuropathic and neuromuscular symptoms (Krumholz 2023). For example, Abicic et al. (2022) recently described oculomotor weakness with diplopia that improved after administration of prednisolone and an AChE inhibitor (Abicic et al. 2022). In 2021, He et al. presented their studies on the sequence analysis of epitopes of the SARS-CoV-2 SGP that are suitable as immunogenic components of potential vaccines. Among 66 peptides, the authors identified 16 suitable gene loci based on their steric position, cytokine release ability, non-toxicity and non-allergenicity using mutational and immunoinformatics analyses (He 2021). Taking into account the recognizability of both HLA-I and HLA-II molecules as well as B- and T-cell relevance, three epitopes (aa 371–387, aa 379–395 and aa 897–913) remained as suitable candidates (He 2021). Two of these sequences correspond exactly to the gene locus (aa 381–386) of SGP, where the PRRA sequence (Farsalinos 2020) described by Changuex et al. (2020) is located, which in turn binds to the extracellular domain of nAChRs (aa 189–192), the "core of the toxin binding site" (Farsalinos 2020). This strongly suggests that vaccines developed using these immunogenic sequences may exhibit the nAChR-blocking effect described by Changeux et al. (2020). The correlations described provide a very plausible rationale for the comparability of symptoms of PVS and offer the possibility of destigmatizing people with PVS (Turner et al. 2023) and offering a promising therapeutic approach instead.

### Reactivation of previous viruses

Numerous studies have shown that a variety of previous or persistent viral diseases (Altmann et al. 2023) (Epstein-Barr virus [EBV] (Altmann et al. 2023), cytomegalovirus [CMV](Altmann et al. 2023), human immunodeficiency virus [HIV] (Peluso 2023) and influenza A virus [IAV] (Kim 2023), infectious bronchitis virus [IBV](Kim 2023), varicella zoster virus [VZV](Kim 2023), human herpesvirus [HHV-1(Kim 2023), HHV-2(Kim 2023), HHV-6 (Kim 2023), HHV-7 (Kim 2023), HHV-8 (Kim 2023)], HBV(Kim 2023) and parvovirus B19 (Kim 2023) may be reactivated in the context of COVID-19 infection or in association with LC. Coincidently, there is now a consensus that not only SARS-CoV-2 can bind to nAChRs. Neri and colleagues (1990) demonstrated high sequence homology between the AAS of HIV-1 gp120 and several AChR-affine snake venom neurotoxins and the nAChR-affine RABV glycoprotein (Lentz et al. 1983; Neri et al. 1990) and demonstrated nAChR binding of HIV-1 (Neri et al. 1990). Bracci et al. (1997) confirmed the high AAS sequence homology of aa160-170 of HIV-1 gp120 and aa189-199 of RABV glycoprotein with the AAS aa30-40 in snake venom and also showed that antibodies formed after RABV vaccination cross-reacted with HIV-1, further confirming the homologous immunity of these AChR high-affinity epitopes (Bracci 1997). In addition, Myles et al. (2003) demonstrated that transcutaneous continuous nicotine application leads to HSV-1 reactivation as measured by viral load in the tear film of latently infected New Zealand White rabbits (Myles 2003). Wang et al. (2024) recently demonstrated that increasing the presence of Dα6 (the insect-associated analog of human α7nAChR) reduced the titer of Drosophila melanogaster (DMG) sigmoid virus (DMelSV) (Wang 2024). Interestingly, the authors were able to reverse this effect with low doses of the low-dose nAChR-antagonistic spinosad (an insecticide), while high doses of spinosad (a high-dose nAChR agonist) in turn led to a reduction in viral load. This bidirectional regulation of virus levels was not possible in Dα6 knockout flies (Wang 2024). According to the allosteric configuration of nAChRs with an increase in LBS after agonistic stimulation (Kenakin 2024; Spurny 2015), this effect can be interpreted as an increase or decrease in nAChR LBS after agonistic and antagonistic stimulation with a corresponding change in nAChR virus binding and thus the detectability of DMelSV. Similarly, Diao et al. (2018) showed an increase in viral load of chronic bee paralysis virus (CBPV) in Italian bees after exposure to high doses of the irreversibly binding (i.e. blocking) nAChR agonist imidacloprid (Diao 2018). Di Pisco et al. (2013) describe identical observations regarding the detectability of Deformed Wing Virus (DWV) after 30 days of sublethal doses of thiamethoxan (irreversible nAChR antagonist) and imidacloprid (Di 2013). The higher binding affinity or concentration of SARS-CoV-2 SGP at nAChRs may outcompete other viral particles, potentially triggering latent viral reactivation.

### Nicotine

Nicotine, identified in 1928 by Posselt and Reimann as an alkaloid component of the tobacco plant (Posselt 1829), makes up approximately 0.6–3% of the dry weight of tobacco plants and is the addictive component of tobacco products (Prochaska 2016). Serum nicotine levels, and thus the clinical effects of nicotine, are highly dependent on the route of administration (Benowitz 2009). The affinity of nicotine for nAChRs is significantly higher than that of ACh and depends on the subunit composition of the receptors, with the inhibition constant (K_i_) of nicotine for α7nAChRs (Leitzke 2023; Gotti 1994) being approximately 30-fold lower (inversely proportional to the binding affinity [Ramsay 2017]) than that of the natural ligand (ACh). The high dissociation constant (pKa = 8.0) explains the large differences in absorption capacity across membranes in different parts of the body. Inhalation of tobacco smoke (pH 5.5—6.02) (Benowitz 2009) results in rapid arterial nicotine uptake (Molyneux 2004) and reaches high concentrations in the ventral tegmental area (VTA) (Le Houezec 2003) within 10–20 s. This leads to high dopamine release in the nucleus accumbens (Yildiz 2004), which explains the behavioral reinforcing effects of smoking (Benowitz 1990) and activation of the reward system (Arias-Carrión et al. 2010). The conformational changes (Buisson 2001) and upregulation of nAChRs (Buisson 2001; Govind 2012) further support the development of addiction and withdrawal symptoms during smoking cessation (Benowitz 1990). All available nicotine replacement therapies (NRTs) are based on venous absorption of nicotine (Wadgave 2016). This results in a significant delay in the rise in concentration and a delayed dopaminergic response in the CNS (Wang 2014). Due to nicotine-induced monoamine oxidase A and B, which degrade dopamine (Sandhu 2024), the delayed absorption is also followed by significantly lower CNS concentrations of the alkaloid (West 2000). CNS nicotine concentrations comparable to smoking have been observed with nicotine nasal spray alone (Guthrie 1999). In their review, Dautzenberg et al. (2021) analyzed a total of 33 studies on the use of transdermal nicotine application in nonsmokers or ex-smokers (Dautzenberg et al. 2021). None of the studies found withdrawal symptoms or associated addictive behavior. In non-smokers, neither craving for inhaled nicotine nor use of another nicotine source after discontinuation of therapy was observed during a longer follow-up period of 12 weeks after discontinuation of transdermal nicotine application (Pullan 1994). Therefore, it can be concluded that transdermal nicotine delivery can be considered safe with respect to the risk of addiction. Base nicotine is readily absorbed transdermally (Benowitz 2009). All available transdermal therapeutic systems (TTS) are based on the release of the active substance through a matrix and/or membrane, which makes the systemic absorption dependent on the release properties of the matrix/membrane and the absorption properties of the skin (i.e. permeability, blood flow). In the liver, nicotine is metabolized to six primary metabolites. Of these, the neuroactive alkaloid cotinine is the most important at 70–80% (Benowitz 2009). The first metabolic steps are catalyzed by CYP2A6 and a cytoplasmic aldehyde oxidase (Benowitz 2009). Nicotine clearance is subject to several influences. For example, the postprandial increase in hepatic perfusion (30%) also results in increased nicotine clearance (40%) (Benowitz 2009). Nicotine clearance decreases by an average of 23% after the age of 65 years (Molander 2001), while the age group between 18 and 69 years shows identical steady-state plasma levels and clearance rates after transcutaneous nicotine application at identical doses (Gourlay and Benowitz 1996). Using urine and blood concentrations of cotinine, Dempsey et al. (2000) showed that metabolic rates are identical in adults, children, and even neonates (Dempsey 2000). In contrast, nicotine and cotinine clearance is significantly higher in women than in men (Benowitz et al. 2006). This difference is increased by oral contraception (28% and 30%, respectively) (Benowitz et al. 2006) and pregnancy (60% and 140%, respectively) (Dempsey 2002) but not by the menstrual cycle (follicular vs. luteal phase) (Hukkanen et al. 2005). Some drugs, such as rifampicin, dexamethasone, and phenobarbital, induce CYP2A6 with considerable interindividual variability (Meunier 2000). Other drugs, such as methoxsalen (8-methoxypsoralen), tranylcypromine, tryptamine, and coumarin, inhibit CYP2A6 (Zhang et al. 2001). Raloxifene, on the other hand, is a potent inhibitor of aldehyde oxidase and therefore potentially interferes with hepatic cotinine formation at the level of nicotinic Δ1′(5′)iminium ions (Obach 2004). However, in clinical practice, only methoxsalen (a phototherapy adjuvant for psoriasis) and tranylcypromine (a monoamine oxidase inhibitor) have been shown to significantly inhibit nicotine clearance in humans (Sellers 2000; Sellers 2003). Ethnicity plays a role only in that people of Black/African ancestry have a slightly lower metabolic rate than all other ethnic groups (total clearance 0.57 vs. 0.76 ml min^−1^ kg^−1^) (Pérez-Stable 1998). The nicotine replacement therapies (NRTs) used produce approximately one to two thirds of the plasma nicotine levels measured in smokers, which range from 10 to 37 ng ml^−1^ (peak concentrations between 19 and 50 ng ml^−1^) (Patterson 2003) and between 10 and 20 ng ml^−1^in TTS (Schneider et al. 2001). Due to tissue distribution, plasma nicotine levels decrease within the next 20 min after smoking with a half-life of 8 min (Benowitz et al. 1988). In nicotine-naïve individuals, the most common immediate mild side effects of nicotine are occasional coughing/sneezing and scratchy throat (Mündel 2017) and the most common severe side effects are tachycardia, nausea, dizziness (Mündel 2017) and diarrhea (Debnath 2022). Occasional mood effects (anxiety [Ouagazzal et al. 1999]), sadness, confusion, anger, energy, and fatigue (Korhonen et al. 2006) have been attributed primarily to nAChRs with α4, α7, or β2 subunits (Tucci et al. 2003). Sleep disturbances (Dependence Update Panel, Liaisons 2000), dizziness (Mündel 2017) as well as headaches (Prochaska 2016) and local skin reactions (Yildiz 2004) have also been observed. At low doses, nicotine has a general depressant effect, while at higher doses it has stimulant effects (Fagerström 2014). In animal studies, nicotine improved vigilance (Lloyd 2000), locomotor activity (Lloyd 2000), cognition in general (Lloyd 2000; Barr 2008), respiratory function (Lloyd 2000), cortical blood flow (Lloyd 2000), EEG activity (Lloyd 2000), and pain tolerance (Lloyd 2000) with improved gastrointestinal and cardiovascular regulation (Lloyd 2000). The originally postulated very low therapeutic range (LD50 0.5–1 mg kg^−1^) has been found to be significantly higher in recent studies, especially with regard to the lethal dose (6.6–13 mg kg^−1^) (Mayer 2014). For NRTs, there is no mention in the literature of life-threatening complications at commercially available doses (Moore 2009). NRTs are even considered safe in patients with stable coronary artery disease (Molyneux 2004), whereas caution is advised in the period (2 weeks) after acute myocardial infarction 2008, in unstable acute coronary artery disease (Molyneux 2004); (2008), during pregnancy (Molyneux 2004), during lactation (Molyneux 2004), and in persons younger than 18 years (Molyneux 2004).

Many cardiovascular pathologies are undoubtedly a consequence of smoking. These include alterations in hemostasis such as increased serum titers of activated platelets (Nair et al. 2001) with increased aggregation and thrombus formation, increased myocardial stress with coronary vasoconstriction (Keeley 1996) and unphysiological serum carbon monoxide concentrations with increased myocardial oxygen debt (Smith 2001). At the same time, proinflammatory and oxidative damage, together with endothelial dysfunction, lead to overall vascular pathology (Ambrose 2004).

In comparative studies between smoking and TTS with respect to platelet activation and increased serum fibrinogen levels observed in smokers, Benowitz et al. (1993) showed that these alterations in hemostasis are not detectable in TTS (Benowitz 1993), which has been confirmed in subsequent studies (Blann et al. 1997); Mundal 1995). The sympathomimetic autonomic imbalance associated with smoking, particularly with respect to autonomic cardiovascular control, is significantly less with transdermal administration of the monosubstance nicotine than with smoking (Lucini et al. 1998). Tobacco smoke contains 80 carcinogens (Li and Hecht 2022). For example, tobacco-associated nitrosamines (TSNA) or polycyclic hydrocarbons are known to be responsible for the development of many malignancies associated with long-term smoking (Church 2009; Bouvard 2009). And although a possible role for nicotine as a co-carcinogen remains controversial (Lee et al. 2011; Mousa^2006^), there is now a large body of data showing that nicotine itself is not carcinogenic (Murray 2009; Murray 1996).

Finally, the UK National Institute for Health and Care Excellence (NICE) concluded in 2013 that "studies with up to five years of follow-up suggest that 'pure' nicotine, as contained in nicotine replacement products (NRT), does not pose a significant health risk" (Lee and Fariss 2016; Mali 2019). Following our hypothesis (Leitzke 2023), many people with LC/ME/CFS have used LDTN to improve their symptoms and have shared their experiences in various forums. The results of a 60-question survey in one of these forums are appended to this paper (Supplement 1) and summarized in the discussion section. In addition, the effect of LDTN on the availability of free LBS of nAChRs was correlated with the course of LC symptoms in a patient under clinical conditions in an imaging experiment.

### Case report

A teacher suffering from persistent symptoms for over three years following SARS-CoV-2 infection was referred to us from the Long COVID Outpatient Department at the University of Leipzig Medical Centre. The most prominent symptom was an inability to speak fluently, which the patient described as a delayed transmission of her perceived normal word-forming impulses to the motor speech-forming organs. The patient was asked to rate her symptoms on a chart (0—none, 1—mild, 2—marked, 3—severe, 4—very severe, and 5—unbearable). Her self-reported symptoms included post-exertional malaise (PEM) (2), fatigue (3), cognitive overstimulation and concentration problems (brain fog) (1), cold extremities (3), dizziness (3), numb fingers (1), and burning or irritated eyes (1).

The hypothesis of viral blockade of nAChRs as a potential cause of her complaints, the therapeutic approach using LDTN and the evaluation of receptor occupancy with molecular hybrid PET imaging using a nAChR-specific tracer ([-]-[^18^F]Flubatine) were thoroughly explained to the patient. She consented to the procedure and the publication of her case details. Continuous LDTN therapy (7 mg/24 h) was administered for 7 consecutive days (days 2–8).

We used the highly selective PET radiotracer (-)-[^18^F]Flubatine for imaging and in vivo quantification of α4β2* nAChRs availability (Sabri 2015; Patt 2013).

Two PET scans within 26 days (day1 and day 27) were performed on a PET/CT with long axial field of view (106 cm, Biograph Vision Quadra, Siemens Healthineers, Erlangen, Germany) in ultra-high sensitivity mode following a bolus injection of 296.2 MBq (pre LDTN) and 298.0 MBq (post LDTN), respectively, MBq (-)-[^18^F]Flubatine consisting of three dynamic acquisitions 0 – 90 min, 195 – 225 min and 330 – 360 min p.i. alongside arterial blood samples were taken over the six hours for the analysis of plasma to whole blood ratios, metabolites as well as plasma to protein binding (Sabri 2015; Patt 2013). To exclude the previously described immediate effect of nicotine on receptor density (Bhatt 2018), the second PET-CT/MRI scan was performed 7 days after LDTN cessation (the half-life of nicotine: 1–2 h (Sanner and Grimsrud 2015) and the absence of the alkaloid was confirmed by a negative cotinine test (Brody 2014).

CT data were used for subject-specific multiple-organ objective segmentation (Sundar 2022), while brain segmentation was performed using FastSurfer (248). Dynamic PET data were motion corrected (Shiyam 2023) and kinetic modeling of the whole body including the brain was performed based on the metabolites-corrected arterial input function and by applying Logan (1990) graphical analysis (t* = 10 min) to generate parametric images of total distribution volume (V_T_), which is defined by the ratio of the concentration of the radioligand in a region of tissue to that in plasma at equilibrium. The tissue itself may contain radioligand that is specifically bound to receptors, nonspecifically bound, or free in tissue water (Innis 2007). The total distribution volume is a linear function of the receptor binding site density available for the tracer and correlates well with the known distribution of α4β2* nAChRs in the brain (Sabri 2015). A detailed description of the kinetic model and its parameters, which was used to describe the PET-tracer dynamic, is given in (Becker 2013).

## Results

Metabolization of the tracer was minimal, with approximately 92% of the unchanged parent compound remaining at 90 min and 91% at 330 min post-injection (p.i.).

**Fig. 2:**
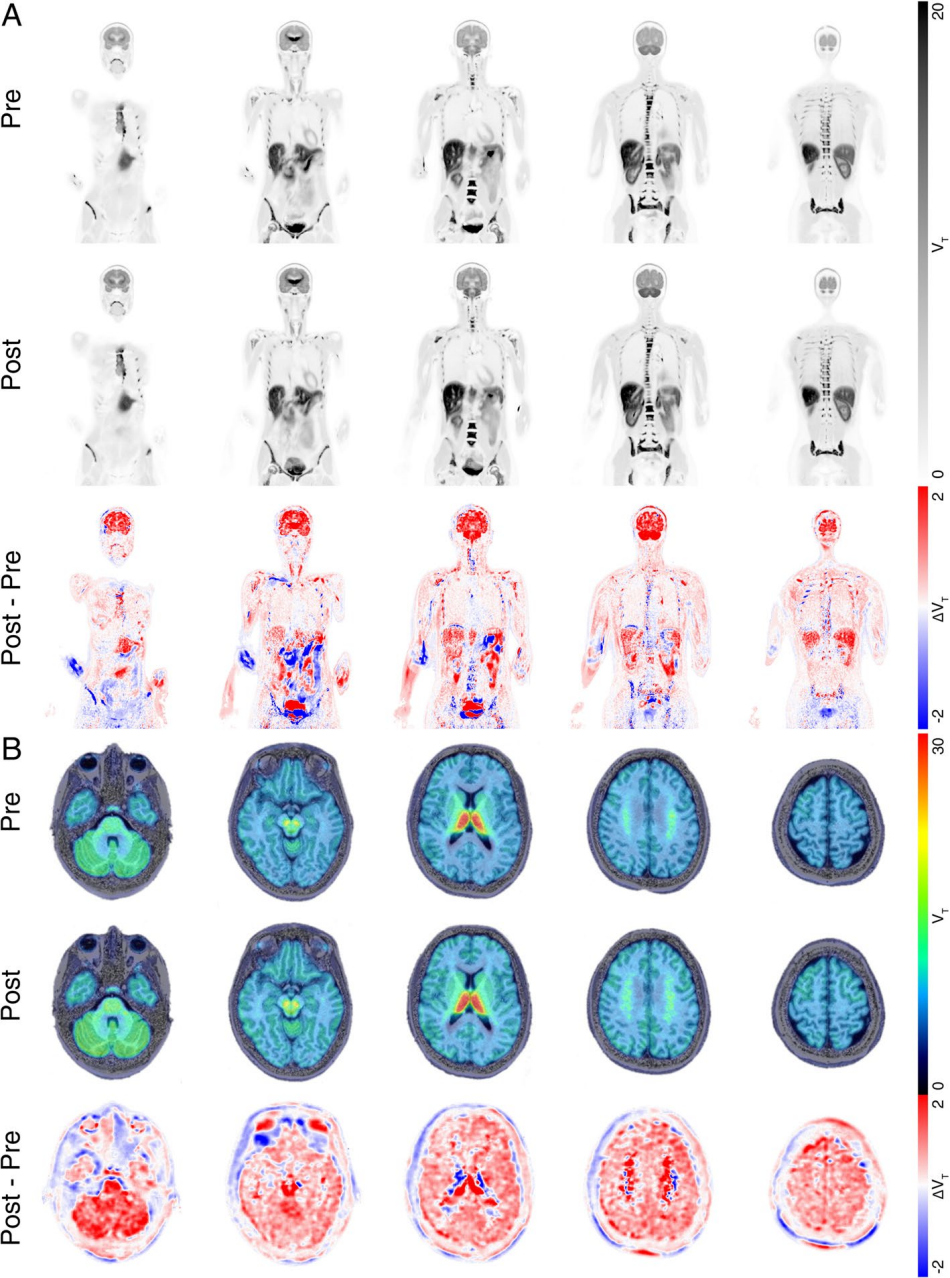
**A** Coronal slices of whole-body (-)-[^18^F]Flubatine PET and **B** axial slices the brain fused with T1-MR images of the same patient before (top row) and after treatment (middle row) with nicotine patches. Parametric images of total distribution volume (obtained by Logan graphical analysis) depict the distribution of α4β2* nAChRs in the body. The higher V_T_ signal observed post-treatment reflects increased receptor availability for the tracer. Bottom row illustrates the absolute V_T_ differences between both scanning sessions with red indicating an increase and blue color a decrease after treatment

Figure 2 shows coronal slices of parametric V_T_ images of both, the pre and post treatment PET scan illustrating a slight increase in V_T_ signal after treatment. This increase was approximately 7.6% within the brain, and −10.7 (upper lobe) – 8.9% (lower lobe) in the lung, 7.4% in the muscles, 5.6% in the liver and 39.2% in the vertebrae (more details can be found in the Supplementary Table 2).

By day 29, the patient's subjective complaints were reduced to dizziness (1) and irritated eyes (1). All the other complaints were no longer detectable.

### Neurological findings

The patient, a 44-year-old woman with a 3.5-year history of nonspecific subjective complaints, underwent specialized investigations. Upon admission, she underwent a comprehensive clinical evaluation, including psychiatric referral. Based on standardized procedures (physical exam, psychiatric exam, structured clinical interview [SCID] I and II) she was diagnosed with functional neurologic disorder (FND) (dysarthria) according to DSM V (F44.4 ICD-10).

After the intervention, her condition was fully resolved. She was subsequently diagnosed with an adjustment disorder (DSM V / F43.1 ICD-10) not reaching the level of major depressive episode (Hamilton Depression Scale [HAMD] 4 points).

At baseline she was rated with CGI-S score of 5 while her follow-up CGI-I score of 2 reflected significant clinical improvement. Concerning CGI, it was developed for use in NIMH-sponsored clinical trials to provide a brief, stand-alone assessment of the clinician's view of the patient's global functioning prior to and after initiating a study medication (Busner 2007). The Clinical Global Impression (CGI) scale was developed for use in NIMH-sponsored clinical trials and provides a brief, clinician-determined assessment of a patient’s global functioning before and after treatment (Busner 2007). It integrates all available information, including the patient’s history, psychosocial circumstances, symptoms, behavior, and functional impact of symptoms. Notably, the CGI-I score determined through clinical judgment at baseline and follow-up aligned with the patient’s self-reported improvements in subjective impairment (Busner 2007). The cognitive recovery (i.e. in speech) might be explained by central nAChR changes alone; however, the disease may also involve other organs or systems such as bone marrow or lymphocytes according to or as indicated by the figure and the review text.

## Discussion

In their study, Godellas, Cymes, and Grosman (2022) challenged the nicotine hypothesis presented by Changuex et al. (2020). Their study tested nicotine’s affinity for only a limited number of α7nAChR AASs on HEK-293 cells, a limitation acknowledged by the authors themselves (Godellas et al. 2022). Other studies have clearly confirmed such an interaction between the SARS-CoV-2 SGP and the LBS of the different nAChRs (Farsalinos 2020; Lagoumintzis 2020). Furthermore, in a previous in silico study using the molecular mechanics Poisson-Boltzmann surface area (MM-PBSA) approach, the α7nAChR fraction had significantly lower binding energy to SARS-CoV-2 compared to α4β2nAChR and αβγδnAChR (muscle-type receptor from the electric organ of Tetronarce californica) (Oliveira 2021), which indicates a lower affinity and may be the reason for lower detectability in the results of Godellas, Cymes, and Grosman (Godellas et al. 2022). Although O´Brien et al. (2023) have demonstrated an augmentation of the effect of PAMs at the nAChRs in whole-cell and single-channel recordings following exposure to SGP (O’Brien et al. 2023) fragments (Chrestia et al. 2022), this phenomenon was only discernible at minimal SGP concentrations. At elevated SGP concentrations, the effect transitioned into an inhibitory mode of operation (O’Brien et al. 2023; Chrestia et al. 2022). It is imperative to underscore that, contingent upon their stoichiometry—defined as the distinct composition of different α- and β-subunits—nAChRs manifest disparate ED_50_ and I_max_ properties with regard to their affinity to agonists, as well as their Ca^++^ conductance. Thus, nAChRs are classified into two distinct categories: high-sensitivity (HS) and low-sensitivity (LS) nAChRs. Furthermore, depending on the receptor occupancy at the corresponding orthosteric (e.g., ACh/nicotine) or allosteric (i.e., NAM/PAM) LBSs, they are characterized by both autologous (ACh) and heterologous (NAMs or PAMs) allosteric conformational changes. This enables nAChRs to adopt different functional states with respect to Ca^++^conductance (open/closed state) and affinity to the corresponding ligands (sensitized/desensitized state), depending on LBS saturation (Papke 2020). This highly complex balance of different functional states is likely to influence the LC/PASC pathology itself, but certainly also the efficacy of therapy with high-affinity ligands.

The findings of the present case study demonstrate an overall augmentation of V_T_ by 7.6% subsequent to the implementation of LDTN. This observation aligns with the discrepancies observed in specific regions of interest among patients diagnosed with mild Alzheimer's disease compared to those categorized as healthy individuals (1–10%) (Sabri 2018), indicating significant impairment in overall brain function.

The studies by Swank et al. (2023) demonstrate the persistence of SARS-CoV-2 associated antigens (S1, full-length SGP, nucleocapsid), indicating the presence of a reservoir in the organism of LC/PASC patients (Swank 2023). These findings support our hypothesis of the persistence of SARS-CoV-2 in binding to nAChRs.

The fact that anthelmintic ivermectin (Shahbaznejad 2021) and the phosphodiesterase inhibitor pyridostigmine (Mestinon) (Davis et al. 2023) alleviate the symptoms in acute COVID-19 and LC patients, respectively is clear evidence that impaired cholinergic transmission is a central problem in LC. Since, ivermectin, as a PAM, increases the conductance of the nAChR cation channel by binding to the allosteric binding site (ABS) of nAChRs and thus enhances the effect of ACh on its receptors (Spurny 2015), mestinon generally increases the concentration of the transmitter at the nAChRs by inhibiting the cleavage of ACh (Pakala 2024), both drugs have a supportive effect on nAChR related cholinergic transmissibility. However, both substances only exert this supporting effect on nAChRs that do not exhibit blockade of the orthosteric binding site (OBS) of these receptors, since ivermectin as a PAM has no competitive effect at the OBS of nAChRs (Spurny 2015) and therefore cannot displace the blocking SGP from this position. Also, increasing the concentration of ACh to OBS (Gotti 1994), due to its high inhibition constant (K_i_) and low affinity, respectively, is not able to remove the viral blockade from the OBS. Thus, the application of the two substances leads to a general improvement of cholinergic transmission at receptors not affected by the blockade in the manner described, but without reversing the blockade of the affected nAChRs, which we believe is causative for LC. It is therefore unlikely that either substance alone will bring about a lasting improvement in the LC problem. Nevertheless, both approaches, in conjunction with vagal nerve stimulation (VNS) via non-invasive techniques (e.g., auricular vagus nerve stimulation (Zheng 2024), coherent breathing (Corrado 2022) or implantable devices (Rangon 2025), may potentially augment the unblocking effect of nicotine. In most cases, a delayed effect of alkaloid application is to be expected. For example, the average half-life of IgG antibodies (pAAs) in the circulation is 3 months (Seijsing 2018).

Additionally, T and B lymphocytes are particularly vulnerable to receptor blockade due to their extremely high number of nAChRs per cell (Skok et al. 2005). This means that an impaired immune response to released SGP is to be expected. In addition, the fact that every human cell could be affected by AChR blockade and that each of these cells has a myriad of AChRs (up to 15,000/cell (Skok et al. 2005)), which in turn exhibit different numbers of LBS (OBS or ABS) as part of allosteric conformational changes, correlates with the unimaginable individuality of the clinical picture of LC. At the same time, transcutaneous nicotine application is also subject to large variations in the number of affected nAChRs and their pharmacokinetic accessibility by nicotine. Consequently, it is almost impossible to predict the effectiveness of SGP elimination and thus the individual course of improvement.

Moreover, the study by Changeux et al. (2020) also showed a sequence homology between the PRRA motif of the SGP and the receptor binding domain of various muscarinic toxins (e.g., cobratoxin, muscarin toxin-like protein) (Changeux 2020). This leaves the question of a possible viral blockade of muscarinic acetylcholine receptors (mAChRs) unanswered. If these acetylcholine receptors are also blocked in the context of LC pathology, further investigations must show whether specific ligands with high affinity to mAChRs (i.e., scopolamine [hyoscine], which is also available for transcutaneous administration) are able to release mAChRs from potential viral binding in correspondence with nicotine in nAChRs. The continuous transcutaneous application of the alkaloid seems to be without alternative, because the presumed large affinity difference between ACh, SGP and the alkaloid in combination with the short half-life of nicotine (Yildiz 2004) makes a rapid re-attachment of SGP to the AChRs after short-term nicotine exposure very likely and possibly causes an SGP cyclic reattachment effect on the nAChRs with recurrence or postponement of symptoms. The reactivation of various previous or latent viral diseases in the context of SARS-CoV-2/LC and the observation that patients with SARS-CoV-2-independent diseases (e.g. ME/CFS, fibromyalgia) were also able to achieve significant improvements in their disease symptoms through transcutaneous nicotine application (see supplement 1) demonstrate the necessity of expanding the evaluation of prospective studies for transcutaneous nicotine application on the clinical picture of LC is of the utmost importance. The studies of Peng et al. (2023) show a significantly increased risk of numerous ADs in association with LC (Peng 2023). As there is no known pathogenetic origin for any of the mentioned incidences (Table 2), which have long been known independently of LC, we would like to discuss the possibility of a post-viral genesis of numerous ADs based on B-lymphocytic nAChR blockade. Further research into this possibility would be associated with the development of causal treatment options for a large number of previously symptomatically treated ADs and should be pursued. This is of extraordinary international importance in view of the high number of people affected, the expected costs in terms of the duration of current symptomatic treatment approaches, but also in view of the long-term professional and social non-participation of patients. In the case presented we saw a clear increase in nAChR LBS after LDTN therapy together with a profound clinical improvement. In particular, the speech formation was fully restored, allowing the teacher to resume her profession without any impairment.

The study, which was limited by the single-case nature of the investigation, supports the hypothesis that the alkaloid nicotine is able to displace SGP from blocked nAChR binding sites and restore proper cholinergic neurotransmission. This might be the central issue in LC/ME/CFS/PVS. In addition, the results of the survey (Supplement 1), although retrospective and not randomized controlled, show clear support for the underlying hypothesis with improvement in 73.5% of cases and reported remissions in a third of cases.

## Conclusions

A review of the literature indicates that a significant disruption of cholinergic neurotransmission might be a central issue for both LC/ME/CFS and PVS. The hypothesis of a viral blockade of nAChRs and the possibility of a competitive reversal of this blockade by LDTN has been corroborated by highly promising results in the broad application of this method to numerous patients. Randomized controlled trials are necessary to determine whether these preliminary results can be substantiated by evidence. However, LDTN application provides many patients with a method that offers a high probability of symptom relief with only minor side effects and represents an affordable therapeutic intervention for the majority of people affected worldwide. Furthermore, dose-finding studies are required to develop individually adapted therapy regimens with regard to dosage and duration of therapy.

## Supplementary Information

Supplementary Material 1.

Supplementary Material 2.

Supplementary Material 3.

## Data Availability

All data utilized in the creation of the case report is available for review in the supplementary materials or may be requested in raw format from the authors.
