# Supplementary material for: Long COVID – a critical disruption of cholinergic neurotransmission?
Source: Bioelectron Med. 2025 Feb 27;11:5. doi: 10.1186/s42234-025-00167-8 (PMC11866872; doi:10.1186/s42234-025-00167-8)
Supplement: Supplementary file 2 — Supplementary Material 2. [file 42234_2025_167_MOESM2_ESM.pdf]

region of interest (ROI)	Pre treatment	Post treatment	Change [%]	Change [VT]
Brain	8,61	9,25	7,61	0,65
Cardiac	2,51	2,54	0,39	0,03
Trachea	2,00	1,52	-24,10	-0,48
Small bowel	4,18	4,63	10,76	0,45
Duodenum	8,90	4,51	-49,27	-4,38
Colon	3,62	2,75	-23,89	-0,86
Urinary bladder	16,88	13,64	-19,22	-3,24
Lung upper lobe	2,45	2,18	-10,73	-0,26
Lung lower lobe	3,23	3,52	8,94	0,29
Lung middle lobe	2,47	2,46	-0,53	-0,01
Muscles	2,91	3,09	7,35	0,18
Spleen	12,03	14,55	20,93	2,52
Kidney	11,68	11,79	0,81	0,10
Gallbladder	7,14	8,70	21,74	1,55
Liver	13,41	14,16	5,57	0,75
Stomach	10,88	9,76	-10,30	-1,12
Pancreas	11,36	9,77	-14,00	-1,59
Adrenal gland	16,01	15,00	-5,91	-1,01
Peripheral bones	2,28	2,19	-12,20	-0,01
Ribs	3,55	4,77	43,10	1,22
Vertebrae	6,81	9,00	39,15	2,19
