# Supplementary material for: Long COVID – a critical disruption of cholinergic neurotransmission?
Source: Bioelectron Med. 2025 Feb 27;11:5. doi: 10.1186/s42234-025-00167-8 (PMC11866872; doi:10.1186/s42234-025-00167-8)
Supplement: Supplementary file 3 — Supplementary Material 3. [file 42234_2025_167_MOESM3_ESM.docx]

# Supplement

Statistical analysis of the questionnaire

Low-dose Transdermal Nicotine (LDTN)

survey

# Abstract

**Background**

Patients from the LongCovid (LC) and the ME/CFS(ME) communities, driven by necessity and guided by personal experimentation, are leading a grassroots transformation of medical research. This new “Natural Behavioral Research” model bridges the gap between patients, clinicians, and researchers and is inspired by behavioural economics championed by Nobel Laureate Daniel Kahneman and others. This new “Behavioural Research” model in medicine will serve to connect knowledge from specialized clinicians and ever increasingly specialized medical specialties or “silos” which currently abandon chronic disease sufferers in a medical no-man’s land. This open model of retrospective data collection is also aimed at harm reduction in a community that has been left without guidance. We aimed to collect data from patients to evaluate the effectiveness, safety and the optimal protocols of low-dose transdermal nicotine patches in both the LC and the ME communities.

# Material and Methods:

Data was collected retrospectively through an online survey with 60 questions after the testers (n=231) consented to anonymous sharing of the data for research purposes (one person declined and was not counted). The survey had a total of 245 responses; however, 14 incomplete responses were removed before analysis. The cohort is representative of the Long Covid/ME/CFS communities (sex, age, and gender). The primary measure is the testers' perceived quantitative change in their Bells’ quality-of-life score from 0% to 100% after completing at least one round of nicotine patches and a break period. Remission events were a secondary measure. Remission events represent short periods (from six to 48 hours) of sickness remission where the patient feels “normal/healthy” (**level 1),** “better than normal” **(level 1+),** or euphoria **(level 2).** For statistical analysis we applied non-parametric Wilcoxon test for comparison of longitudinal data and the non-parametric Kruskal-Wallis test for group comparisons of three or more groups. Fisher’s exact test was used to test for differences in distribution of count data. Associations were tested using either the Spearman correlation coefficient (ordinal data) or Pearson correlation coefficient (continuous data).

# Results:

Nearly three out of four of the testers (73.5%) reported an improvement in baseline (see Figure 1), with approximately one in three experiencing remission events (levels 1,

1+, and 2), either during or shortly after discontinuing nicotine patch use (see Figure 3). The important variable of sex did not show differences in response.

Longer treatment duration and older age correlate with increased benefit (see Figure 2). Figure 4 shows non-parametric correlations found significant associations between the amount of different experienced side effects and age (Spearman correlation, p < 0.05). Lastly, regardless of smoking status, an increase in Bell’s Baseline after treatment is significant in all smoking levels (Wilcoxon, p < 0.05). Furthermore, the level of increase in baseline does not statistically differ by the smoking status (Kruskal-Wallis, p > 0.05).

The complete set of anonymous data in Excel format is available upon request.

# Conclusions:

Although retrospective, this data shows a clear clinical benefit for LDTN as a treatment for LC and ME. The broad benefit for both long-term ME and LC sufferers also gives weight to the hypothesis that the underlying disease is cholinergic dysfunction. Although short-lived, the remission events also give hope to a population that has felt abandoned for years/decades. The ability for testers to self-manage dosing also improves safety and outcomes. Increased benefits with age may be related to the age-related cognitive benefits of nicotine patches which is being evaluated in the ongoing Mind study for Alzheimer’s disease. Weight to dose also hints at a trend to better results with lower dose-per-kilo regimens. This may be due to the dose and duration dependent effects of nicotine. The dose and length of treatment differences appear in the data since non-responders stop quickly which skews the data. The reported symptoms analysis also hints at subgroups in the LC & ME communities, but more research with prospective longitudinal data will be needed to confirm phenotyping by symptoms and response to LDTN.

Demographic table

| **Group size [n]** | 231 |
| --- | --- |
| **Gender [n]** | 61 male  166 female  4 other |
| **Age [years]** | 46.3 ± 12.8 |
| **Weight [kg]** | 73.5 ± 16.6 |
| **Smoking status [n]** | 158 Non-smoker  66 Past-smoker  7 current smoker |
| **Time of nicotine patch**  **application [days]** | 17.8 ± 8.6 |
| **Syndrome [n]** | 117 Long Covid  47 Long Covid and ME/CFS 59 ME/CFS  5 Coronavirus Vaccine Side Effects 3 other |
| **Fibromyalgia [n]** | 215 no  16 yes |

Values are given as mean ± standard deviation

1. Does low-dose transdermal nicotine improve symptoms?
2. for Long COVID (LC)
3. for ME/CFS


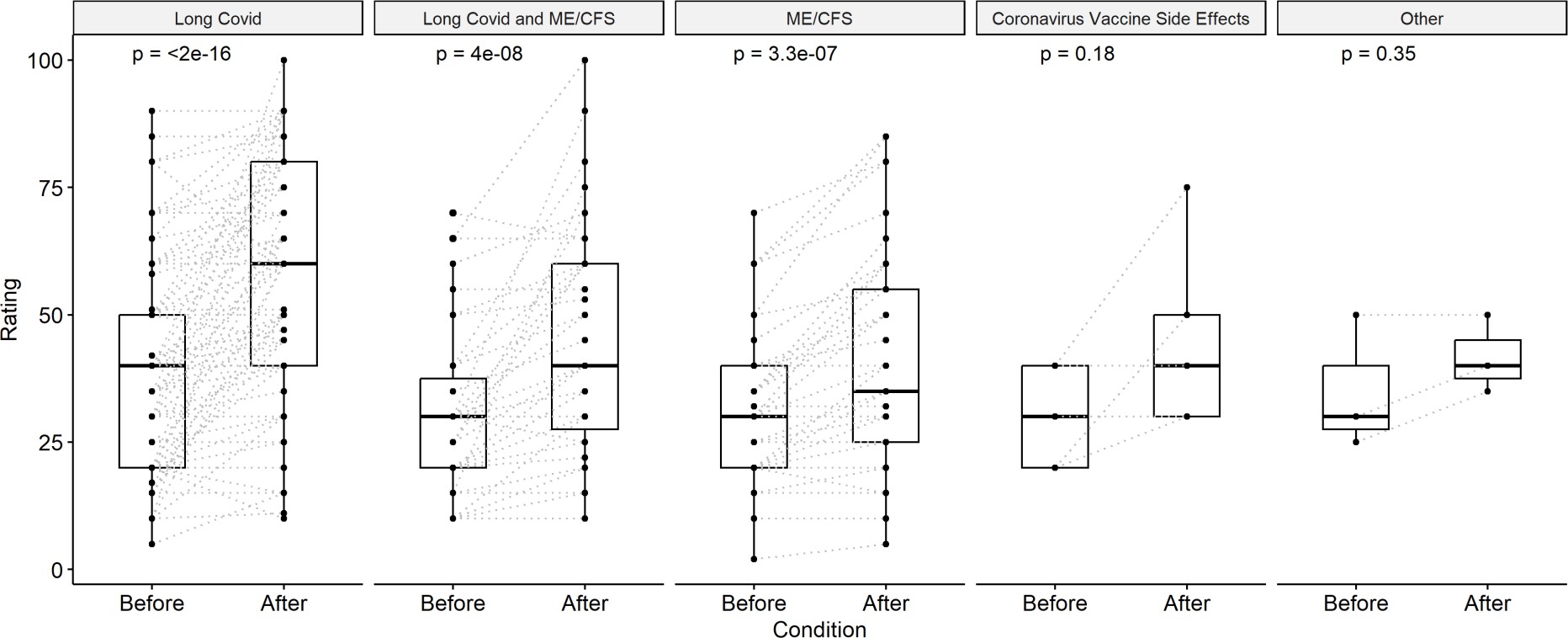


Yes, the three subgroups „Long Covid“, „Long Covid and ME/CFS“ and „ME/CFS“ show significant changes after treatment with an increase in Bell’s score baseline QOL ratings (Wilcoxon, p < 0.05).

The subgroups „Coronavirus Vaccine Side Effects“ and „Other“ also improved but the changes are not statistically significant because the groups were too small.

Descriptive table of mean ratings and distribution of responders after treatment:

| Syndrome | Rating | | Responder | | |
| --- | --- | --- | --- | --- | --- |
|  | Before | After | Decrease | Stable | Increase |
| Long Covid | 39.5 ± 19.0 | 56.1 ± 22.3 | 4 (3.4 %) | 20  (17.1 %) | 93  (79.5 %) |
| Long Covid and ME/CFS | 30.3 ± 15.9 | 45.2 ± 21.5 | 2 (4.3 %) | 5 (10.6 %) | 40  (85.1 %) |
| ME/CFS | 31.3 ± 14.1 | 40.1 ± 20.1 | 1 (1.7 %) | 24  (40.7 %) | 34  (57.6 %) |
| Coronavirus Vaccine Side  Effects | 30 ± 10 | 45 ± 18.7 | 0 (0 %) | 2 (40 %) | 3 (60 %) |
| Other | 35 ± 13.2 | 41.7 ± 7.6 | 0 (0 %) | 1 (33.3 %) | 2 (66.7 %) |

Rating is given as mean ± standard deviation; Responder is given as amount of patients per syndrome with proportion in percent.

Fisher’s exact test found a significant difference in the distribution of the count data between syndrome and responders, i.e. the rate of responders is not homogeneous distributed for all syndromes (p = 0.007).

1. Fibromyalgia


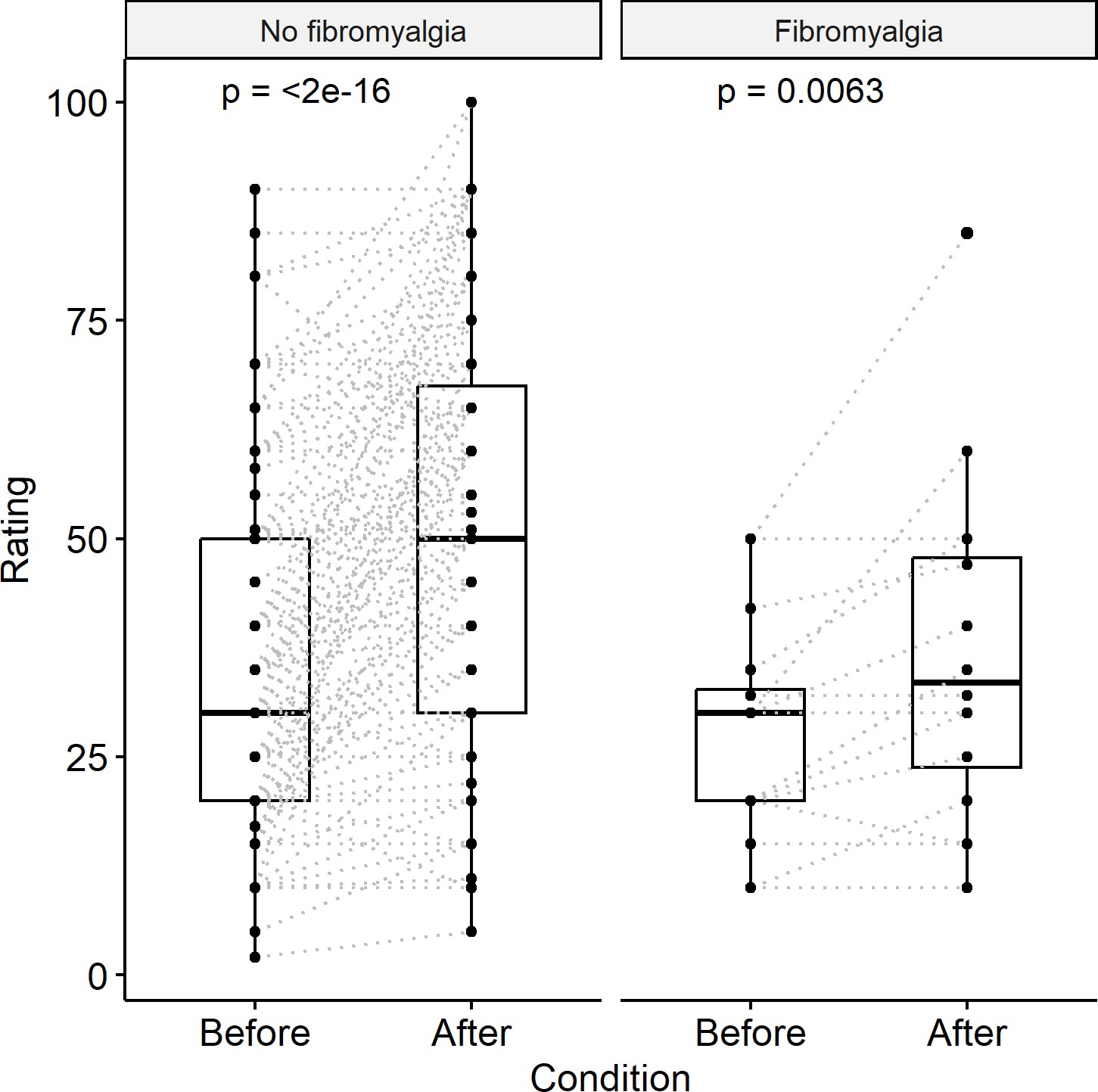


Both subgroups, patients with and without fibromyalgia showed significant changes with an increase in their rating after treatment (Wilcoxon, p < 0.05).

|  | Rating | | Responder | | |
| --- | --- | --- | --- | --- | --- |
|  | Before | After | Decrease | Stable | Increase |
| No fibromyalgia | 35.9 ± 17.7 | 50.3 ± 22.4 | 6 (2.8 %) | 47 (21.9 %) | 162 (75.3 %) |
| Fibromyalgia | 27.8 ± 12.4 | 36.5 ± 19.2 | 1 (6.25 %) | 5 (31.25 %) | 10 (62.5 %) |

Rating is given as mean ± standard deviation; Responder is given as amount of patients per syndrome with proportion in percent.

The distribution of the count data between fibromyalgia syndrome and responders does not differ (Fisher’s exact test, p = 0.27).

1. Are there any variables in terms of efficiency?
2. age
3. dose/weight
4. duration of use/number of cycles
5. gender


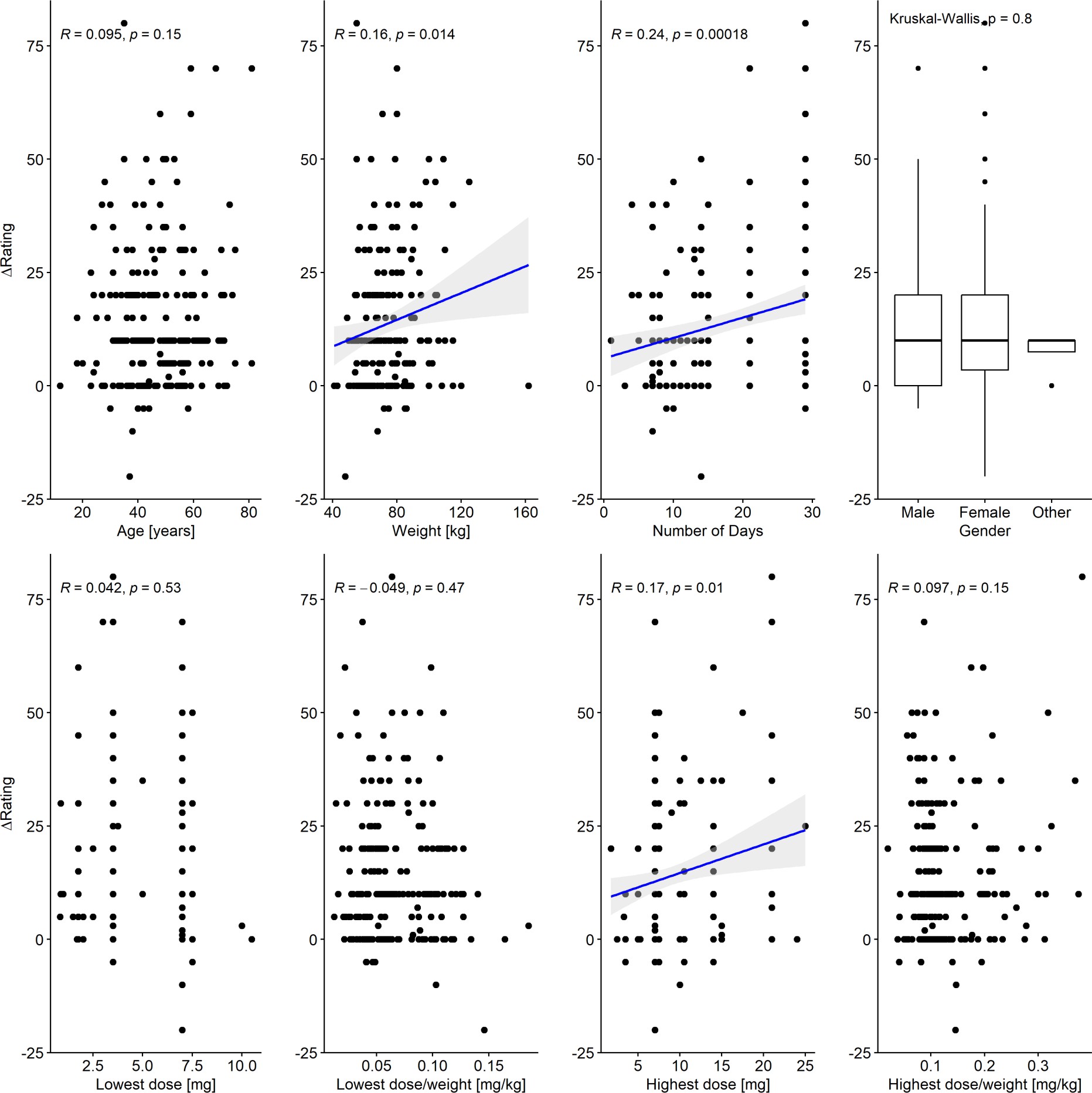


There was a significant association between the change in Bell’s Baseline Score after treatment with nicotine patches and the patient’s weight, the duration of application (number of days) and highest applied dose (Pearson correlation, p < 0.05). In contrast, age, gender, lowest applied dose as well as dose-weight-ratio do not seem to be related to self-reported improvements after treatment (p > 0.05).

1. Are there short term remission events?
   1. Variables (as in 2. a,b,c,d) shown here


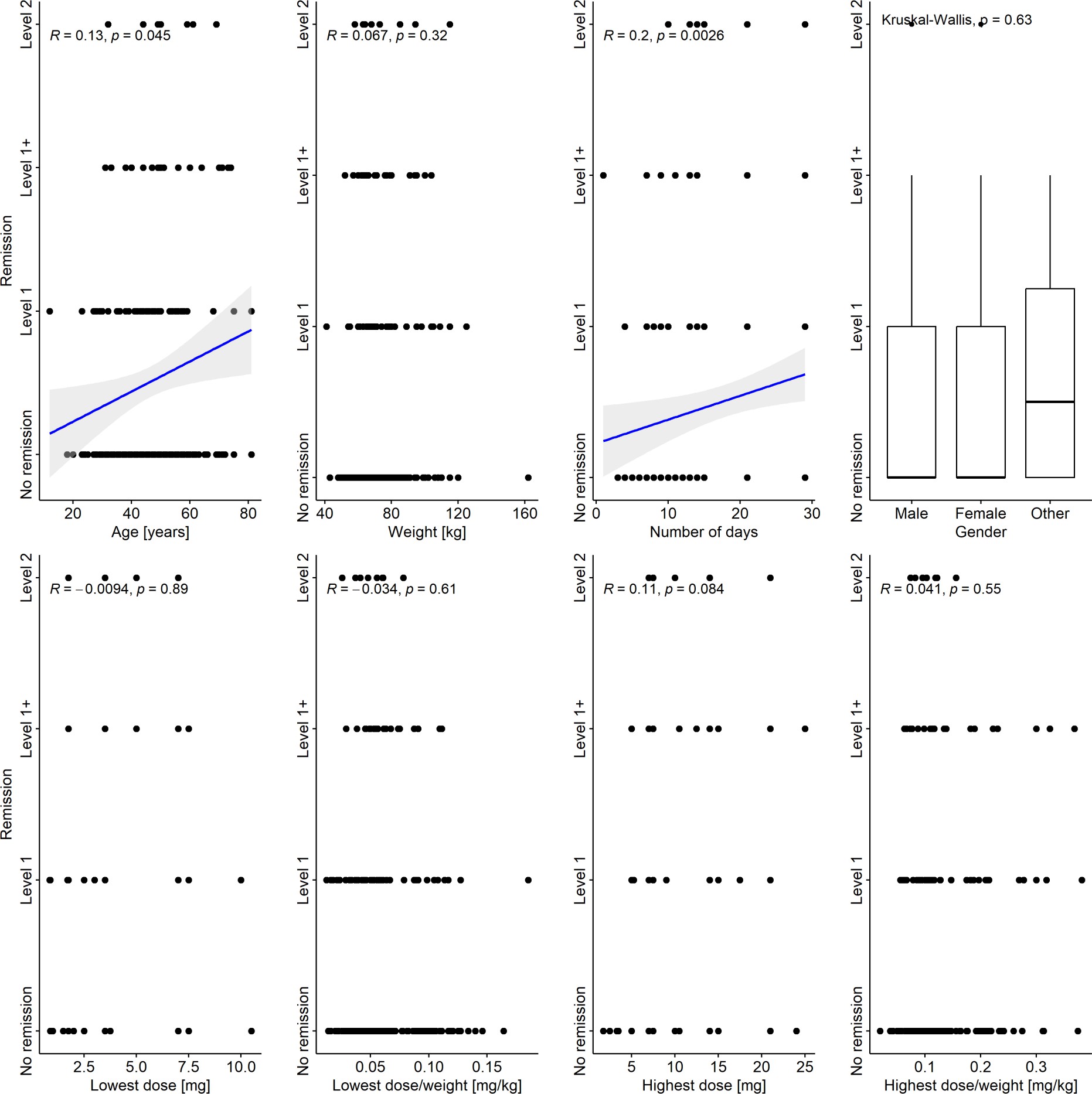


Non-parametric correlation analysis found significant associations between level of remission and age as well as number of days using nicotinic patches (Spearman correlation, p < 0.05). All other measures do not relate to the levels of remission (p > 0.05).

|  | Responder | | |
| --- | --- | --- | --- |
|  | Decrease | Stable | Increase |
| No Remission | 7 (4.5 %) | 46 (29.7 %) | 102 (65.8 %) |
| Level 1 | 0 (0 %) | 3 (6.7 %) | 42 (93.3 %) |
| Level 1+ | 0 (0 %) | 2 (9.5 %) | 19 (90.5 %) |
| Level 2 | 0 (0 %) | 1 (10 %) | 9 (90 %) |

Responder is given as amount of patients per remission level with proportion in percent.

Fisher’s exact test found significant differences in distribution of the count data between levels of remission and responders, i.e. the rate of responders is not homogeneously distributed for all levels of remission (p = 0.003). That is confirmed by a significant correlation between remission

levels and change in rating before and after treatment (Spearman correlation R = 0.43, p = 7.4e- 12).


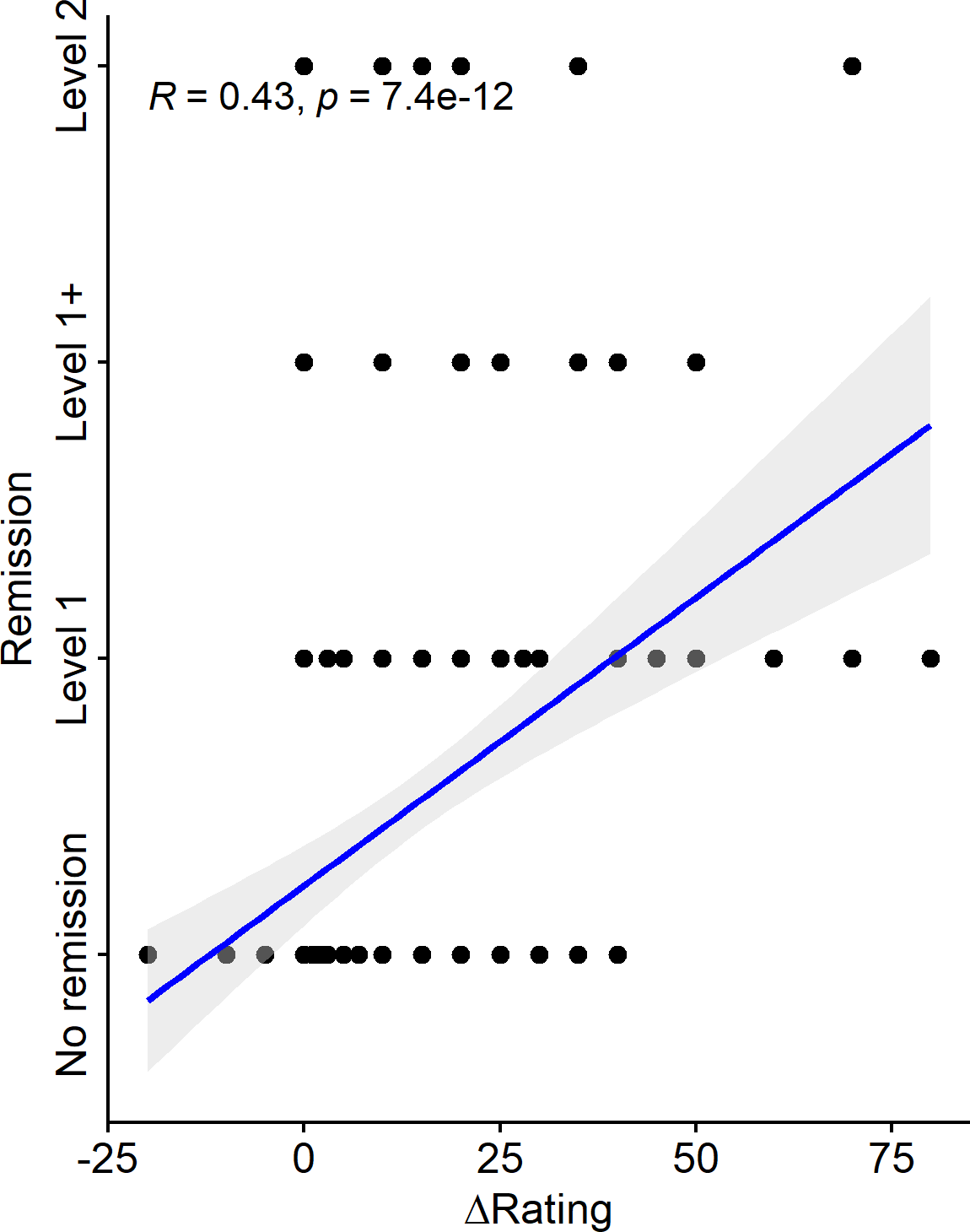


1. Are there side effects?
   1. dependencies (as in 2.a,b,c,d) can be shown here


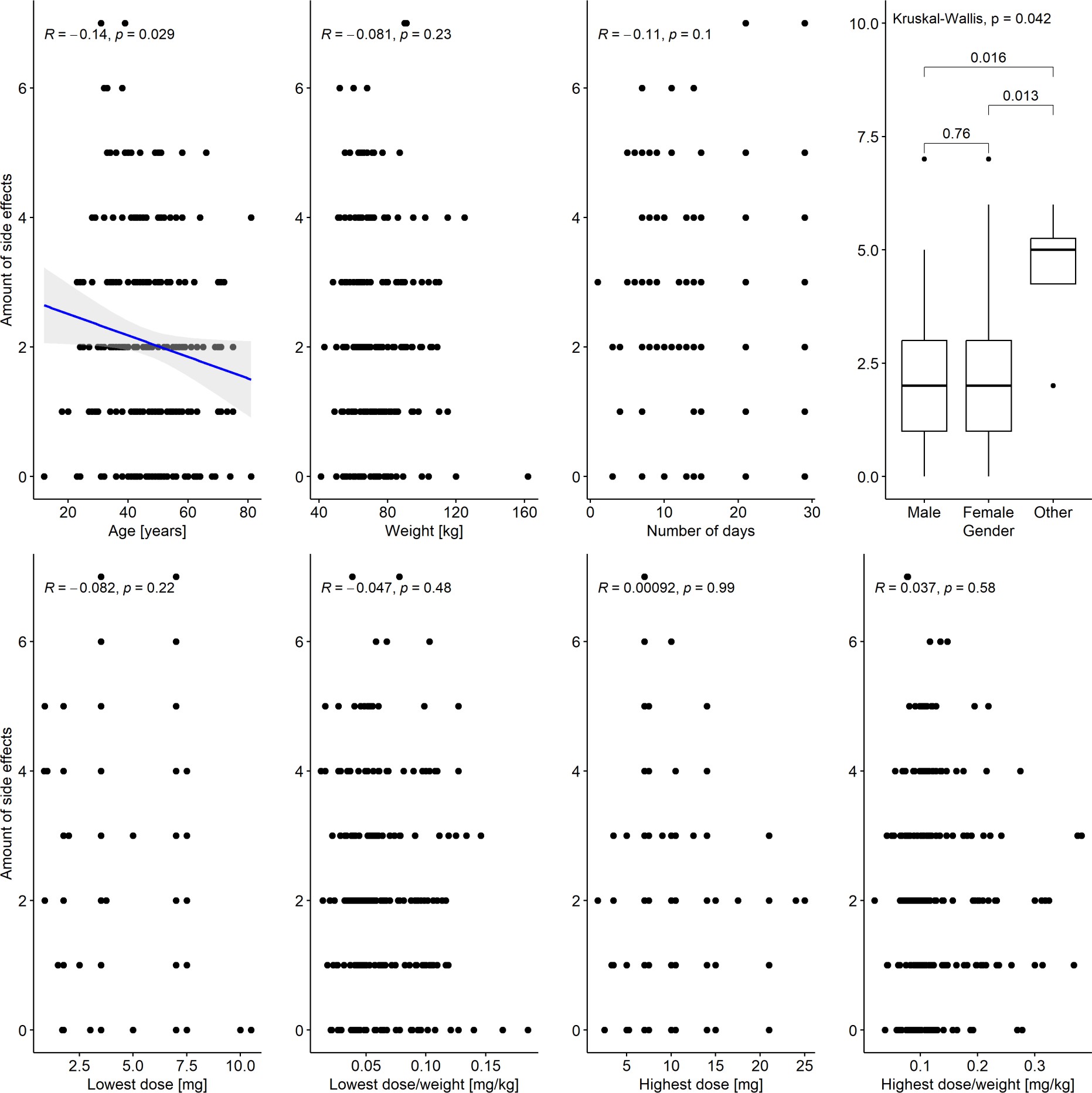


Non-parametric correlations found significant associations between amount of different experienced side effects and age (Spearman correlation, p < 0.05). In addition, patients who describe themselves as non-binary experienced significantly more side effects (Kruskal-Wallis, p < 0.05). All other measures do not relate to the amount of side effects (p > 0.05).

|  | Responder | | |
| --- | --- | --- | --- |
|  | Decrease | Stable | Increase |
| No side effects | 0 (0 %) | 5 (19.2 %) | 21 (80.8 %) |
| Side effects | 7 (3.4 ) | 47 (22.9 %) | 151 (73.7 %) |
| Herx/Verx | 6 (6.6 %) | 15 (16.5 %) | 70 (76.9 %) |
| Nausea | 5 (5.1 %) | 24 (24.2 %) | 70 (70.7 %) |
| Diarrhea | 2 (4.5 %) | 10 (22.7 %) | 32 (72.7 %) |
| Sleep disturbance | 6 (5.3 %) | 31 (27.2 %) | 77 (67.5 %) |

| Tachycardia | 4 (8.2 %) | 11 (22.4 %) | 34 (69.4 %) |
| --- | --- | --- | --- |
| Loss of appetite | 0 (0 %) | 9 (22.5 %) | 31 (77.5 %) |
| Brain fog | 2 (9.1 %) | 6 (27.3 %) | 14 (63.6 %) |
| Sadness | 1 (5 %) | 5 (25 %) | 14 (70 %) |

Responder is given as amount of patients per side effect with proportion in percent.

The distribution of the count data between side effects (yes/no) and responders does not seem to differ (Fisher’s exact test, p = 0.92), i.e. experiencing or noting no side effects seems independent of status of treatment response. That is also reflected by a missing correlation between the amount of side effects and change in rating before and after treatment (Spearman correlation R = -.12, p = 0.064, data not shown).

1. Smoking history


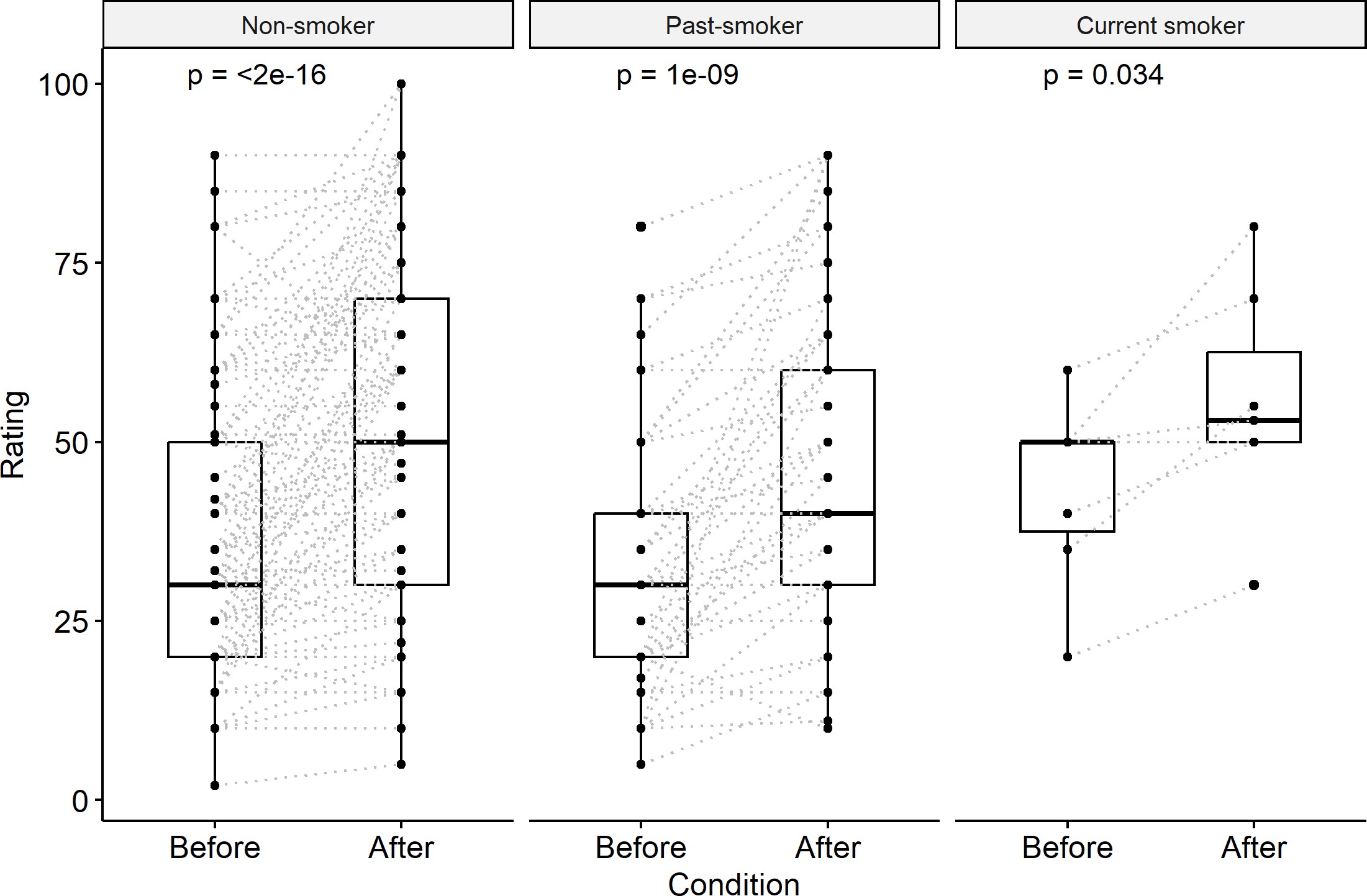


An increase in Bell’s Baseline after treatment is significant in all smoking levels, i.e. independent of smoking status (Wilcoxon, p < 0.05). Furthermore, the level of increase does not statistically differ by the smoking status (Kruskal-Wallis, p > 0.05).

|  | Rating | | Responder | | |
| --- | --- | --- | --- | --- | --- |
|  | Before | After | Decrease | Stable | Increase |
| Non-smoker | 36.3 ± 17.9 | 50.5 ± 23.2 | 6 (3.8 %) | 36 (22.8 %) | 116 (73.4 %) |
| Past-smoker | 31.9 ± 16.4 | 46 ± 20.9 | 1 (1.5 %) | 15 (22.7 %) | 50 (75.8 %) |
| Current smoker | 43.6 ± 13.1 | 55.4 ± 16 | 0 (0 %) | 1 (14.3 %) | 6 (85.7 %) |

Rating is given as mean ± standard deviation; Responder is given as amount of patients per syndrome with proportion in percent.

The distribution of the count data between smoking status and responders does not differ (Fisher’s exact test, p = 0.91).
